# Substantial Heritability Underlies Fairness Norm Adaptation Capability and its Neural Basis

**DOI:** 10.1002/advs.202411070

**Published:** 2024-12-16

**Authors:** Yuening Jin, Dang Zheng, Ruolei Gu, Qingchen Fan, Martin Dietz, Changshuo Wang, Xinying Li, Jie Chen, Yuanyuan Hu, Yuan Zhou

**Affiliations:** ^1^ CAS Key Laboratory of Behavioral Science Institute of Psychology Chinese Academy of Sciences Beijing 100101 China; ^2^ Department of Psychology University of Chinese Academy of Sciences Beijing 100049 China; ^3^ Department of Early Childhood Education China National Children's Center Beijing 100035 China; ^4^ Center of Functionally Integrative Neuroscience Institute of Clinical Medicine Aarhus University Universitetsbyen 3 Aarhus 8000 Denmark; ^5^ Sino‐Danish Center University of Chinese Academy of Sciences Beijing 100049 China; ^6^ Brainnetome Center Institute of Automation Chinese Academy of Sciences Beijing 100190 China; ^7^ CAS Key Laboratory of Mental Health Institute of Psychology Chinese Academy of Sciences Beijing 100101 China; ^8^ The National Clinical Research Center for Mental Disorders & Beijing Key Laboratory of Mental Disorders Beijing Anding Hospital Capital Medical University Beijing China

**Keywords:** anterior insula, fairness norm adaptation, genetics, prediction error encoding, supplementary motor area, twin studies

## Abstract

The present research uncovers the shared genetic underpinnings of fairness norm adaptation capability, its neural correlates, and long‐term mental health outcomes. One hundred and eighty‐six twins are recruited and played as responders in the Ultimatum Game (UG) while undergoing fMRI scanning in their early adulthood (Study‐1) and are measured on depressive symptoms eight years later (Study‐2). With computational modeling, the process of norm adaptation is differentiated from that of fairness valuation in UG. The two processes both have moderate levels of heritability. The anterior insula has a significant phenotypic correlation, whereas the Supplementary Motor Area/Medial Frontal Gyrus (SMA/mSFG) shows both a significant phenotypic correlation and a shared genetic influence with the learning rate, an index for norm adaptation. The dopaminergic *DRD2* polymorphisms correlate with both the learning rate and the SMA/mSFG encoding of prediction error, constituting of their common genetic basis. Regional gene expression analysis reveals the high expression of dopamine‐related genes in the SMA/mSFG. Moreover, the learning rate can predict depressive symptom severity eight years later, with the *DRD2* polymorphisms constituting their shared genetic basis. This suggests that heritability is a non‐negligible driving force behind norm adaptation, which facilitates the learning of social norms in changing environments and preserves long‐term mental health.

## Graphical abstract

Heritability is a non‐negligible driving force behind learning of social norms and its neural basis, which enhances the rapid acquisition of adaptive values across generations, granting an evolutionary advantage in changing environments in terms of maintaining long‐term mental health. *DRD2* polymorphisms constitute the shared genetic basis of norm learning capability, its neural basis, and long‐term mental health.

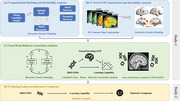

## 1 Introduction

The Cultural Evolution Theory posits that stability and change are two major ways through which social norms evolve.^[^ ^1^, ^2^ ^]^ While adaptive norms endure across generations, maladaptive norms are supplanted by new norms through a social learning process to ensure viability in altered social conditions.^[^ ^1^, ^2^ ^]^ The field has already realized the role of heritability in fueling one dynamic of norm evolution, namely norm stability.^[^ ^1^, ^2^ ^]^ This implies that norms crucial for survival are readily passed down through generations.^[^ ^3^ ^]^ This phenomenon expedites our evolution into a modern society that inherently upholds social norms of reciprocity and obligation. Could heritability fuel the other dynamic of norm evolution, namely norm changes as well? Norm changes essentially rely on the rapid acquisition of adaptive norms to occur. Despite its profound survival implications, the heritability of norm acquisition capability is scarcely investigated. The rapid acquisition of adaptive norms (i.e., high social learning capability) passed down across generations could grant us an evolutionary advantage similar to the transmission of adaptive norms themselves. Only heritability fuels both sides of norm dynamics could we both quickly develop and preserve adaptive norms and acquire a survival advantage in changing environments.

Fairness norm, being one of the most prevalent type of social norms, exhibits significant variability across situations, which makes the issue of norm learning more pronounced.^[^ ^4^ ^]^ A strong capability to learn what constitutes an acceptable fair offer in different time periods from different stakeholders holds profound survival implications.^[^ ^5^, ^6^, ^7^ ^]^ On the one hand, in non‐challenging times, enforcing norms against malicious transgressors safeguards our self‐interests, preserves our dignity, and deters potential transgressors. On the other hand, in challenging times, timely adjustment of norm standards, setting minimal expectations on others, becomes crucial, allowing us to both dissipate negative feelings and accumulate resources offered by stringent allocators. A strong adaptation capability thus grants both resources and psychological wellbeing in the long run. Multiple empirical evidence suggests that heritability might constitute an indispensable force in shaping the capability of learning fairness norm. First, the domain‐general cognitive aspects of social norm learning processes (such as cognitive flexibility) have more than 30% heritability.^[^ ^8^ ^]^ Second, the domain‐specific processes of moral appraisal associated with fairness norm, along with their relevant neural activities, also exhibit more than 30% heritability.^[^ ^9^, ^10^, ^11^, ^12^ ^]^ However, it still needs to determine the heritability of our ability to learn social norms within the fairness domain and its underlying neural basis.

The lack of studies investigating the genetic contribution to fairness norm learning and adaptation can be attributed, in part, to the technical challenges of its quantification. However, recent developments in computational modeling have addressed the technical challenges, allowing for differentiating a norm adaptation process from a fairness valuation process in the fairness decision process in the Ultimatum Game (UG).^[^ ^13^, ^14^, ^15^ ^]^ The system of norm adaptation (indexed by the learning rate), which captures how fast one learns and adjusts one's norm, is both conceptually and neurologically distinct from the system of fairness valuation indexed by the initial fairness norm and fairness sensitivity, which captures norm strictness and sensitivity.^[^ ^13^ ^]^ We are interested in whether the norm adaptation system has a distinct pattern of genetic influence different from the fairness valuation system. Understanding the unique genetic influences underlying these two processes provides deeper insights into how individuals adapt to social norms versus how they appraise unfairness, which ultimately can inform interventions targeting each process and advance research on each of the two aspects of fairness decisions.

After heritability analyses, this study hypothesized that genetic expression related to neurotransmitter activity, particularly the dopamine D2 receptor gene (*DRD2*), which strongly modulates dopaminergic levels, would be associated with norm adaptation ability and the underlying neural activities. Previous studies have documented dopaminergic influence on both fairness‐specific and general learning processes. In the Dictator Game (DG), individuals who play as dictators receive endowments in each round and are asked to allocate a proportion of the endowment to another player. Enhanced dopamine levels increased the dictator's sensitivity to both advantageous and disadvantageous inequity proposals in the DG, reinforcing the representation of social norms in people's minds and prompting them to reduce inequality.^[^ ^16^ ^]^ Additionally, studies have found that enhanced dopamine levels accelerate the learning process by facilitating the minimization of prediction errors (PE), that is, narrowing the gap between actual rewards and expectations.^[^ ^17^ ^]^ This study specifically focuses on three *DRD2* polymorphisms due to their strong influences on the density of *D2* receptors and in turn dopamine levels, and their wide‐recognized impacts on a broad dimension of social behaviors and learning processes.^[^ ^18^ ^]^ For social behaviors, *DRD2* expression has been shown to influence the preference for social novelty, social approach behaviors across species.^[^ ^19^, ^20^ ^]^ A human genome‐wide association study (GWAS) also revealed the association of sociability scores with variation in the gene encoding in *DRD2*.^[^ ^21^ ^]^ For learning processes, previous studies have identified that multiple *DRD2* polymorphisms, linking with *D2* receptor density, were related to individual capabilities in avoiding negative outcomes in probabilistic learning tasks.^[^ ^22^, ^23^ ^]^ Genotypes associated with higher *D2* receptor availability (i.e., the *A2* allele carrier in *rs1800497* and the T/T homozygotes of *rs6277*) have consistently shown superior performance in learning to avoid negative consequences across studies.^[^ ^22^, ^23^ ^]^ Based on these findings, we propose that dopaminergic genes modulating dopamine levels could have an effect on the rate of norm adaptation. We focused on three single nucleotide polymorphisms (SNPs) that strongly influence the density of the *D2* receptor, namely *rs1800497, rs2283265*, and *rs6277*. The prevalence of the *A1* allele (*T*) in both the *DRD2/ANKK1‐Taq Ia (rs1800497)* and *rs2283265* polymorphism leads to a reduction of 30% density of the *D2* receptor compared to the *A2* allele (*C or G*), leading to lower dopamine levels.^[^ ^24^, ^25^ ^]^ The prevalence of T in *DRD2 C957T (rs6277)* leads to an enhancement in the density of striatal *D2* receptor.^[^ ^26^ ^]^ We thus hypothesized that the T allele in *DRD2* genotypes (*rs1800497* and *rs2283265*), and the G allele in *rs6277*, would reduce the learning rate for fairness norms. While the association between dopaminergic neurons and reinforcement signals is well‐established, the role of serotonin in learning processes has been less clear.^[^ ^27^ ^]^ Some evidence suggests that serotonin is involved in controlling impulsivity, that is, inhibition of an impulsive response upon viewing an unfair offer.^[^ ^28^ ^]^ Recent studies have found that serotonin is specifically associated with learning to avoid negative events but does not directly pertain to norm learning.^[^ ^27^ ^]^ We thus hypothesized that only dopaminergic, but not serotonergic SNPs, would influence the learning rate for norms − in other words, a predominant dopaminergic genetic influence on the norm adaptation system.

On the other hand, we hypothesized that the fairness valuation system may be simultaneously modulated by dopaminergic and serotonergic genetic influences. Previous studies have documented the modulation of both the serotonergic and dopaminergic systems on the proposal allocation process in the DG/UG, which involves the detection of fairness violations. Serotonin levels has consistently been negatively associated with rejection rates toward unfair offers in the UG, as observed through experimental manipulations and natural observations in multiple studies.^[^ ^29^, ^30^, ^31^, ^32^ ^]^ It is possible that serotonergic SNPs, which modulate serotonin levels, would influence the fairness valuation system denoted by the strictness of fairness norm and fairness sensitivity in the UG. Among serotonergic SNPs, the most extensively studied influencer of social behavior is the tryptophan hydroxylase‐2 gene (*TPH2, rs4570625)*. This study specifically focuses on the *TPH2* polymorphism due to its wide‐recognized influence on serotonin levels, social behavior, emotional regulation, and cognitive control. Previous studies have found that the T allele in the *rs4570625* generally relates to increased serotonin levels.^[^ ^33^, ^34^ ^]^ For social behavior, the T allele in the *rs4570625* promotes social cooperation among humans.^[^ ^28^, ^35^ ^]^ *TPH2* genetic deactivation models of mice revealed elevated aggressive behaviors.^[^ ^36^ ^]^ For emotional regulation, the influence of *rs4570625* extends to the prediction of trait emotional instability, the occurrence of major depressive disorder (MDD), and negative responses to stressful life events.^[^ ^37^, ^38^, ^39^ ^]^ For cognitive control, accumulative evidence pointed to the association between *TPH2* variants and cognitive control capabilities and neuropsychiatric disorders characterized by cognitive control disabilities.^[^ ^40^ ^]^ The fairness decision in the UG might be subject to the influence of *TPH2* polymorphism since it involves cognitive control and is also emotionally demanding.^[^ ^41^ ^]^ Specifically, we hypothesized that the T allele in the genotype of *rs4570625* would be associated with a less strict (lower) initial fairness norm imposed on proposers and lower fairness sensitivity. The role of the dopaminergic system in modulating fairness decisions has also been well‐documented in previous studies. Higher dopamine levels are generally associated with the willingness to seek for higher personal economic incomes rather than to altruistically punish others.^[^ ^42^ ^]^ Accordingly, we hypothesized that the T allele in *rs1800497* and *rs2283265*, as well as the G allele in *rs6277*, which relates to a reduction in dopamine levels, would be associated with higher (stricter) initial fairness norms and higher fairness sensitivity.

Taking a further step, we investigate the heritability and the dopaminergic genetic modulation of the neural basis of norm adaptation. Learning of fairness norms essentially involves the encoding of the difference between the expected norm and the newly‐encountered split ratio, that is, the encoding of norm PE.^[^ ^14^ ^]^ Previous studies have shown the convergent involvement of the insula and a range of brain regions related to PE encoding, including the medial orbitofrontal cortex, ventromedial prefrontal cortex, and substantia nigra/ventral tegmental area.^[^ ^13^, ^14^, ^15^ ^]^ To date, the genetic influences on the neural encoding of norm PE remain unknown.

Taking another step further, we hope to discover both the phenotypic and common genetic connection between norm adaptation capability, its neural basis, and adaptation consequences about long‐term mental health, to provide preliminary support for our hypothesis that a genetically‐powered adaptation capability would grant us a biological advantage on long‐term survival. A longitudinal design spanning for eight years gives us an opportunity to answer this question.

**Figure 1:**
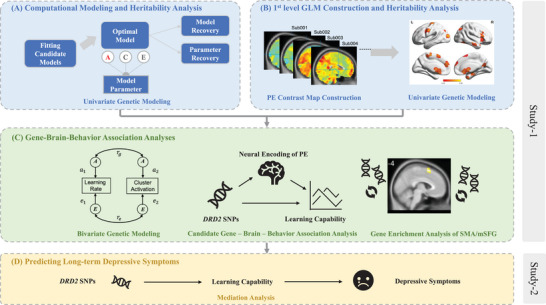
Analytical Procedures. A) Computational modeling of the decision‐making process in the UG and heritability analysis with univariate genetic modeling on key parameter indices (i.e., learning rate, initial fairness norm, and fairness sensitivity) and behavioral indices (i.e., acceptance rate). B) Construction of first‐level GLM and voxel‐wise heritability analysis to identify brain clusters with credible genetic influence. C) Gene–brain‐behavior association analysis: bivariate genetic modeling analysis to identify brain clusters showing both a significant phenotypic and genetic correlation with the learning rate, and mediation analysis to examine the mediation role of neural encoding of PE in SMA/mSFG between *DRD2* polymorphisms and the learning capability, and a gene enrichment analysis of SMA/mSFG to provide supplementary evidence for the high expression of dopaminergic genes in this brain region. D) Learning capability could buffer the occurrence of long‐term depressive symptoms, and then we investigated whether *DRD2* constituted their common genetic basis, and further tested the mediating role of learning capability between DRD2 gene polymorphism and depressive symptoms.

**Figure 2:**
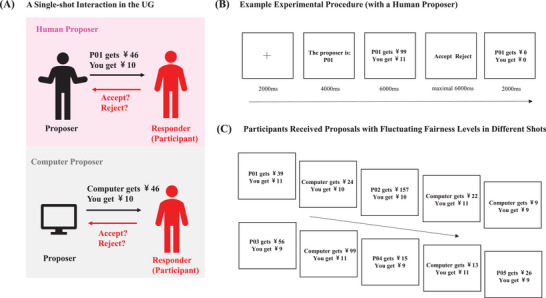
Experimental Procedures. A) In each trial, participants were randomly matched to either an anonymous human proposer or a computer‐generated proposer. Each human proposer was denoted by a random alphanumeric code instead of names or photos to control confounding effects. In the cover story, participants were told that proposals from human proposers were made by real participants, whereas proposals from computer proposers were randomly generated by a computer program. Proposals from human/computer proposers were identical and preset by experimenters. Participants could either accept or reject the proposal by pressing different buttons on an MRI‐compatible button box. The decision “acceptance” resulted in splits according to the proposal, whereas “rejection” resulted in nothing for both players. After they made the decision, the results would be shown on the screen. B) An example experimental procedure for a single shot of the game where participants faced a human proposer. C) Participants received proposals with fluctuating fairness levels from different proposers in different shots of the game.

In brief, the current study endeavors to investigate the genetic basis underlying norm adaptation ability and its neural activities with a relatively large twin sample through a series of analyses across two studies (**Figure** **1**). In Study‐1 (fMRI experimental study), the participants performed a modified version of UG while undergoing functional magnetic resonance imaging (fMRI) (**Figure** **2**). We used the computational modeling approach to separate the adaptation system from the fairness valuation system in the UG and delved into their heritability. Further, we examined whether unique dopaminergic genetic influences are responsible for the norm adaptation system, which could be distinct from the genetic underpinnings of the fairness valuation system. We then used univariate genetic modeling and bivariate genetic modeling analyses to further identify brain regions whose activity, modulated by PE, not only demonstrates phenotypic correlation but also shares common genetic influences with the learning rate. Last, we explored whether dopamine‐related gene activity constitutes the shared genetic foundation, with a particular focus on the candidate *DRD2* genetic polymorphisms. Therefore, complementary to the SNP analyses, we provided additional supporting evidence for the broader dopaminergic expression on brain regions responsible for encoding norm PE signal, drawing from the Allen Human Brain Atlas (AHBA).^[^ ^43^ ^]^ In Study‐2 (longitudinal survey), we recalled the twin cohort 8 years later since study‐1 and traced their depressive symptoms. We investigated whether norm adaptation capability could predict the occurrence of depressive symptoms in the long term and whether these traits shared a common dopaminergic genetic basis.

## 2 Experimental Section

### 2.1 Participants

To ensure an adequate sample size to study the brain–behavior relationship, in Study‐1, 100 pairs of same‐sex twins were initially recruited from the Beijing Twins Brain–Behavior Association Project, which was dependent on the Beijing Twin Study.^[^ ^44^, ^45^ ^]^ Eight participants and their twin siblings in seven pairs (two from the same pair) were excluded due to not understanding the instructions or provided random behavioral responses. This resulted in valid data from 93 pairs of twins (52% female; 48 monozygotic pairs (MZ), 45 dizygotic pairs (DZ)) for behavioral analyses. Their age ranged from 16 to 26 years (mean (*M*) = 20.27; standard deviation (*SD*) = 2.36). Six pairs of twins were furthermore excluded due to excessive head motion (see the subsection *fMRI data acquisition and preprocessing*). This resulted in 87 pairs of twins (54% female; 44 MZ, 43 DZ; age *M *= 20.21; *SD *= 2.24) in the fMRI analyses. In Study‐2, 122 participants were successfully recalled 8 years later in December 2023 and administered BDI‐II, a self‐report measure of depressive symptoms.^[^ ^46^ ^]^ None of the participants had self‐reported current/history of physical/psychiatric diagnoses, current psychiatric medications, neurological or metabolic illnesses, or head injuries. None of the participants had self‐reported family history of psychiatric diagnoses. All participants read and signed the informed consent before the experiment. The study was approved by the Institutional Review Board of the Institute of Psychology, Chinese Academy of Sciences (#H15024 for Study‐1, and #H20037 for Study‐2).

### 2.2 Task and Experimental Design of UG

Participants played a one‐shot anonymous UG in the E‐prime 2.0 environment while undergoing functional magnetic resonance imaging (fMRI).^[^ ^47^ ^]^ Scanning comprised 2 sessions of equal lengths, with a 20‐second break in between. All participants played the role of UG responder in all the trials (Figure 2). Participants were given a financial reward as a token for participation in this study plus remuneration, which would be determined by their actual income from two randomly selected trials. Their choices of acceptance or rejection would influence the proposers’ remuneration as well, which would be distributed to the corresponding players at the end of the experiment.

Participants played 48 trials in total, 24 with human proposers and 24 with computer proposers. A full list of proposal splits is shown in Material S1‐1 (Supporting Information). The amount of money offered to the responder was fixed to one of 9, 10, and 11 yuan. The fairness level was manipulated by varying the ratio distributed to the responder against the sum of money distributed to the proposer and the responder. Twenty‐four types of ratios ranging from 4% to 50% were included. This study adopted a within‐subject design of 2 (proposer type) * 24 (ratio).

### 2.3 Analytical Strategies Overview

The analytical procedures comprised behavioral and neuroimaging analyses (Figure 1). In the behavioral analyses, a model‐free generalized linear mixed‐effects model (GLMM) was first conducted to inspect the key features of behavioral responses, which provided insights into computational model specification. Then candidate models were built, and model selection, model recovery, and parameter recovery were performed. Within the optimal model, heritability analyses were conducted on acceptance rates and model parameter estimates with a univariate genetic modeling approach, to decompose the variance of each variable into the genetic (A, which also stands for heritability), common environment (C), and unique environment (E) components.^[^ ^48^ ^]^ In the neuroimaging analyses, first, a model‐based fMRI analysis was conducted to explore the neural activities of the PE encoding process extracted from the computational model. Specifically, a first‐level GLM was conducted for each participant and calculated the PE contrast map for the human proposer condition for each individual. The heritability of each voxel was then explored in order to find aggregated clusters with significant heritability under certain threshold criteria. To attain this goal, voxel‐wise univariate genetic modeling was conducted to identify voxels with ≥90% posterior probability of having a significant genetic influence in the posterior probability map (PPM). Clusters with size ≥20 were extracted from the PPM. With these clusters extracted, it was to determine whether they have phenotypic and genetic correlations with the learning rate. To attain this goal, the phenotypic correlation and the common genetic influence between the mean activation of these clusters in the PE contrast map and the learning rate were estimated using the bivariate genetic modeling approach. Bivariate genetic modeling allowed the estimation of phenotypic correlation and genetic overlap between two variables.^[^ ^48^ ^]^ To open up the black box of common genetic influence between brain and behavior, the common dopaminergic SNPs constituting of the shared genetic influence between brain and behavior were further identified. To elucidate how learning rate related to long‐term adaptation outcomes, the phenotypic correlation and common genetic basis between learning capabilities and depressive symptoms eight years later with bivariate genetic modeling analysis were further investigated. The association between learning capabilities and other long‐term learning and adaption outcomes was also investigated to provide support for the external validity of the learning rate.

### 2.4 Statistical Analysis

#### 2.4.1 Study‐1 (fMRI Experimental Study)

##### Generalized Linear Mixed Effects Model (GLMM)

First, model‐free analyses were conducted to clarify 1) whether participants had different fairness sensitivity toward human versus computer proposers and 2) whether the acceptance rate evolved with time when participants face human versus computer proposers. If yes for (1), fairness sensitivity should be estimated separately for human and computer proposers in computational models. If yes for (2), time should influence decision outcomes after controlling for proposer type and ratio, which implied a changing fairness norm. A logistic GLMM was used to investigate the main and interactional effect of proposer type, split ratio, and time on the binomial decision outcome of acceptance or rejection with the *lme4* and *emmeans* package in R4.1.3. The random intercept of family and subject was estimated.

##### Computational Modeling

The computational modeling essentially depicted how individuals as responders made the acceptance or rejection decision in each round, by comparing the valuation of the two options. A higher valuation of an option resulted in a higher probability of choosing that option (formula (3)). The valuation of the rejection option was 0 (formula (2)), whereas the valuation of the acceptance option was the weighted sum of monetary utility and fairness concern. Whereas monetary utility related to the monetary gain, fairness concern was essentially a comparison between the proportion of money received in the current round and fairness norm in the mind (i.e., the proportion expected to receive from proposers) (formula (1)). The fairness norm in the mind was learned in the norm adaptation process following a reinforcement learning rule, and the learning rate depicted the speed of learning such fairness norm (formula (4)(5)(6)). Following the examples of previous studies,^[^ ^13^, ^14^, ^15^ ^]^ the above 3 critical processes were quantitatively specified (i.e., a value calculation process, a decision process, and a norm adaptation process). Different candidate norm adaptation models were built with slight differences in the norm adaption process. Model fitting and model comparisons were performed to select the optimal model with the best fit with the experimental data. The specific formula for each process is shown below.

##### Value Calculation Process

Individuals calculated the utility of the acceptance and rejection options to make a decision. Similar to Gu, et al.,^[^ ^13^ ^]^ the utility of acceptance reflected monetary utility ((1  −  β)*r(*responder*)) plus the Fehr–Schmidt (FS) inequality aversion utility ($\beta \;\times \;r\left(responder\right)\;\times \;\left[\frac{r(responder)}{r(proposer)\;+\;r(responder)}\;-\;fairnorm\right]$) (Fehr and Schmidt, 1999) (formula (1)).

(1) $$\begin{array}{ccc} \mathrm{Gain}\left(\mathrm{acce}\mathrm{pt}\right) & = & \left(1-\beta \right)\times r\left(\mathrm{resp}\mathrm{onder}\right)+\beta \times r\left(\mathrm{resp}\mathrm{onder}\right)\\ & & \times \left[\frac{r\left(\mathrm{resp}\mathrm{onder}\right)}{r\left(\mathrm{prop}\mathrm{oser}\right)\;+\;r\left(\mathrm{resp}\mathrm{onder}\right)}-\mathrm{fair}\mathrm{norm}\right] \end{array}$$

Inequality aversion essentially involves a comparison between the split ratio encountered ($\frac{r(responder)}{r(proposer)\;+\;r(responder)}$) and the fairness norm in mind, an internal representation of what constitutes fair (*fairnorm*). This comparison was then transformed to the same scale as the monetary utility by multiplying the amount of money received by the responder (*r*(*responder*)). Then it was weighted by a free parameter, the fairness sensitivity β. The bare monetary gain (*r*(*responder*)) was also weighed by (1  −  β) to 1) keep monetary utility and inequality aversion utility on the same scale and 2) the higher the sensitivity for fairness, the lower the preference for money. Different fairness sensitivity was specified for human and computer proposers (β_*h*_ and β_*c*_) if participants were shown to have different fairness sensitivity toward human versus computer proposers in the GLMM analysis. The utility of rejection was 0, as both players gained nothing (formula (2)).

(2) $$Gain\;\left(rej\right)=0$$

##### Decision Process

A softmax function was used to link the utility of an option with individuals’ probability of choosing that option (formula (3)). Two free parameters, τ and ξ were included. τ depicts the level of randomness in choice. ξ depicts the occasional unintended choice by mistake.^[^ ^49^ ^]^ Both of them represent noise in decisions.

(3) $$\begin{array}{ccc} p\;\left(\mathrm{acc}\right) & = & \xi \times \frac{\exp\left(\tau \times \mathrm{Gain}\left(\mathrm{acc}\right)\right)}{\exp\left(\tau \times \mathrm{Gain}\left(\mathrm{acc}\right)\right)+\exp\left(\tau \times \mathrm{Gain}\left(\mathrm{rej}\right)\right)}\\ & & +\;\left(1-\xi \right)/2 \end{array}$$

##### Candidate Norm Adaptation Processes

Multiple candidate models were built to represent the norm adaptation process. Previous studies consistently showed that participants had an internal representation of the fairness norm, that was, the fairness level to be expected from a virtual human proposer on each trial, and participants could flexibly adjust the norm according to their interaction histories to adapt to the changing environment in the single‐shot UG, similar to the one used in this study.^[^ ^13^, ^14^, ^15^ ^]^ Therefore, it was assumed that the learning of fairness norms occured when participants interacted with human proposers. As previous modeling studies did not include a computer proposer in their experiment, it was assumed that when facing computer proposers, participants had no learning process (formula (4)), or learned at the same rate as human proposers where fairness norm toward human versus computer proposers update separately (formula (5)), update together (formula (6)), learned at a different rate compared with human proposers (formula (7) or update together (formula (8)). The formula represented how individuals adjust their subjective fairness norm based on the prediction error, that is, the difference between the split ratio of the current trial and the pre‐existing norm in their mind. The difference was weighted by the learning rate α, which was a free parameter.

(4) $$\;\mathrm{fair}\mathrm{norm}=\left\{\begin{array}{c} \mathrm{fair}\mathrm{nor}m_{h}+\alpha_{h}\times \left(\frac{r\left(\mathrm{resp}\mathrm{onder}\right)}{r\left(\mathrm{prop}\mathrm{oser}\right)\;+\;r\left(\mathrm{resp}\mathrm{onder}\right)}-\mathrm{fair}\mathrm{nor}m_{h}\right)\;\;\;\;\;\mathrm{for}\;\mathrm{human}\;\mathrm{prop}\mathrm{osers}\\ \mathrm{fair}\mathrm{nor}m_{c}\;\;\;\;\;\mathrm{for}\;\mathrm{comp}\mathrm{uter}\;\mathrm{prop}\mathrm{osers} \end{array}\right.$$

(5) $$\;\mathrm{fair}\mathrm{norm}=\left\{\begin{array}{c} \mathrm{fair}\mathrm{nor}m_{h}+\alpha \times \left(\frac{r\left(\mathrm{resp}\mathrm{onder}\right)}{r\left(\mathrm{prop}\mathrm{oser}\right)\;+\;r\left(\mathrm{resp}\mathrm{onder}\right)}-\mathrm{fair}\mathrm{nor}m_{h}\right)\;\;\;\;\;\mathrm{for}\;\mathrm{human}\;\mathrm{prop}\mathrm{osers}\\ \mathrm{fair}\mathrm{nor}m_{c}+\alpha \times \left(\frac{r\left(\mathrm{resp}\mathrm{onder}\right)}{r\left(\mathrm{prop}\mathrm{oser}\right)\;+\;r\left(\mathrm{resp}\mathrm{onder}\right)}-\mathrm{fair}\mathrm{nor}m_{c}\right)\;\;\;\;\;\mathrm{for}\;\mathrm{comp}\mathrm{uter}\;\mathrm{prop}\mathrm{osers} \end{array}\right.$$

(6) $$\;\mathrm{fair}\mathrm{norm}=\mathrm{fair}\mathrm{norm}+\alpha \times \left(\frac{r\left(\mathrm{resp}\mathrm{onder}\right)}{r\left(\mathrm{prop}\mathrm{oser}\right)\;+\;r\left(\mathrm{resp}\mathrm{onder}\right)}-\mathrm{fair}\mathrm{norm}\right)$$

(7) $$\;\mathrm{fair}\mathrm{norm}=\left\{\begin{array}{c} \mathrm{fair}\mathrm{nor}m_{h}+\alpha_{h}\times \left(\frac{r\left(\mathrm{resp}\mathrm{onder}\right)}{r\left(\mathrm{prop}\mathrm{oser}\right)\;+\;r\left(\mathrm{resp}\mathrm{onder}\right)}-\mathrm{fair}\mathrm{nor}m_{h}\right)\;\;\;\;\;\mathrm{for}\;\mathrm{human}\;\mathrm{prop}\mathrm{osers}\\ \mathrm{fair}\mathrm{nor}m_{c}+\alpha_{c}\times \left(\frac{r\left(\mathrm{resp}\mathrm{onder}\right)}{r\left(\mathrm{prop}\mathrm{oser}\right)\;+\;r\left(\mathrm{resp}\mathrm{onder}\right)}-\mathrm{fair}\mathrm{nor}m_{c}\right)\;\;\;\;\;\;\mathrm{for}\;\mathrm{comp}\mathrm{uter}\;\mathrm{prop}\mathrm{osers} \end{array}\right.$$

(8) $$\mathrm{fair}\mathrm{norm}=\left\{\begin{array}{c} \mathrm{fair}\mathrm{norm}+\alpha_{h}\times \left(\frac{r\left(\mathrm{resp}\mathrm{onder}\right)}{r\left(\mathrm{prop}\mathrm{oser}\right)\;+\;r\left(\mathrm{resp}\mathrm{onder}\right)}-\mathrm{fair}\mathrm{norm}\right)\;\;\;\;\;\mathrm{for}\;\mathrm{human}\;\mathrm{prop}\mathrm{osers}\\ \mathrm{fair}\mathrm{norm}+\alpha_{c}\times \left(\frac{r\left(\mathrm{resp}\mathrm{onder}\right)}{r\left(\mathrm{prop}\mathrm{oser}\right)\;+\;r\left(\mathrm{resp}\mathrm{onder}\right)}-\mathrm{fair}\mathrm{norm}\right)\;\;\;\mathrm{for}\;\mathrm{comp}\mathrm{uter}\;\mathrm{prop}\mathrm{osers} \end{array}\right.$$

The initial fairness norm was specified at the beginning of the UG as a free parameter. In candidate models, either the same or different initial fairness norm was specified for human and computer proposers. This generated 8 candidate models (Table S2‐1, Supporting Information).

The Hierarchical Bayesian estimator in Rstan was used to simultaneously derive group‐level and individual estimates for all participants.^[^ ^50^ ^]^ The ranges and prior distribution for each parameter at the group and individual level are shown in Table S2‐2 (Supporting Information). The goodness of fit was compared using the leave‐one‐out information criteria (LOOIC). LOOIC calculated the pointwise out‐of‐sample prediction accuracy using the log‐likelihood of the simulated posterior parameter values.^[^ ^51^ ^]^ Model recovery was performed to test whether the simulated data from the winning model could capture key characteristics in the original data. Parameter recovery was performed to examine the robustness of parameter estimation (for details, see Material S3, Supporting Information).

##### Heritability Analyses of Behavioral Indicators

To determine the heritability underlying behavioral indicators, the intraclass correlation (ICC) was first calculated and performed genetic modeling analyses on the rejection rate in the UG and key parameters (i.e., initial fairness norm, learning rate, and fairness sensitivity) in the winning computational model, controlling for age and sex. ICC ((*MS_between_*  −  *MS_within_*)/(*MS_between_*  +  *MS_within_*)) measured the proportion of total variance attributable to the differences between the twin pairs.^[^ ^52^ ^]^ MS_between_ and MS_within_ stand for mean squared between and mean squared within, respectively. A significantly higher ICC observed among MZ than DZ derived from the Fisher r‐to‐z transformation potentially indicated a considerable genetic influence on the interested behavior indicator, as the only source of within‐pair similarity for MZ more than DZ came from the additive genetic influence.^[^ ^48^ ^]^ The univariate genetic modeling analysis was then performed with the *OpenMx* package in R3.1.2 to partition the additive genetic (A) (referred to as heritability), shared environmental (C), and non‐shared environmental (E) contributions to variance in behavioral indicators.^[^ ^53^ ^]^ The above variance decomposition in twins data was calculated based on three key assumptions: MZ pairs correlated 1 for A. DZ twin pairs had half degree of correlation for the additive genetic component compared to the MZ twin pairs (DZ correlate 1/2 for A), MZ and DZ twin pairs shared their environments to the same extent (MZ and DZ both correlates 1 for C), and also experienced unique environmental influences that contributed to differences in behavioral indicators (MZ and DZ both correlate 0 of E).^[^ ^48^ ^]^ Both the full ACE model and various sub‐models (i.e., AE, CE, and E) were fitted, and we selected the optimal model referring to Chi‐square changes, AIC, and the principle of parsimony.^[^ ^54^, ^55^ ^]^

##### fMRI Data Acquisition and Preprocessing

Magnetic resonance images were acquired on a 3 Tesla GE Discovery MR750 MRI scanner at the Institute of Psychology, Chinese Academy of Sciences, Beijing. Image acquisition details and preprocessing steps (slice‐timing, realignment, normalization, smoothing) were presented in Material S4 (Supporting Information).

##### First‐level GLM

As norm adaptation entails the computation of prediction errors (PE), first‐level general linear model (GLM) analyses were first conducted to discover brain regions that encode prediction error (PE; the split ratio in the current trial minus the fairness norm in the previous trial) for each individual. Specifically, a parametric analysis was used to identify brain regions modulated by PE. Each GLM included 3 main events: proposal display, decision screen, and outcome display in a one‐factorial (proposer type) design matrix constructed by convolving each event onset with a canonical hemodynamic response function. The PE for each trial extracted from the computational model was entered into the GLM as a parametric regressor at the proposal display event. Residual effects of head motion were accounted for by including the estimated six motion parameters for each subject as covariates. The PE contrast map was then built for each individual at the proposal revelation event in the human proposer condition. The human proposer condition was only included because both GLMM and the optimal model revealed that learning only occurred toward human proposers, but not computer proposers.

##### Voxel‐wise Univariate Genetic Modeling on PE Maps

Voxel‐wise univariate genetic modeling was performed to estimate the genetic, shared environmental, and non‐shared environmental contributions to each voxel in the prediction error (PE) *t*‐maps. For each voxel, the full ACE model and various sub‐models (i.e., AE, CE, and E) were compared based on Chi‐square changes, AIC and the principle of parsimony, and selected an optimal model for each voxel.^[^ ^54^, ^55^ ^]^ One best‐fitting model who has the maximal number of voxels optimally fitting on this model was finally selected.

For almost all the voxels, the best‐fitting model was AE (see the Results section). The significance of A was then determined by comparing the chi‐square with and without A (AE model vs E model) for each voxel. A significant (*p* < 0.05) decrease in chi‐square indicated a significance contribution of A on each voxel. A posterior probability map (PPM) was then constructed for PE to identify regions with ≥90% posterior confidence showing a credible additive genetic effect.^[^ ^12^, ^56^ ^]^ PPM marked the posterior probability for each voxel, that an effect exceeded a particular threshold (a prior mean of zero in SPM) and it enabled Bayesian inference about regionally specified effects.^[^ ^57^ ^]^ Given that it was probability‐based, it thus had no multiple comparison issues. If a voxel exceeded 90% posterior confidence showing a credible additive genetic effect, it was marked 1, and otherwise 0. In this way, a PPM was formed leaving only voxels with a credible genetic effect. From this map, all clusters with voxel sizes greater than 20 were extracted for subsequent analyses as these regions of interest (ROI). Then the average heritability in each of these ROIs was calculated. The averaged parametric estimate value in each of these ROIs was also calculated for each participant for the followed analyses.

##### Bivariate Genetic Modeling

Bivariate genetic modeling was performed for the learning rate and the average parametric estimate in each ROI. With this analysis, brain regions that exhibit significant phenotypic correlation with the learning rate (Bonferroni correction *p* < 0.05 per number of ROIs) could be identified. Following traditional bivariate genetic modeling methods, the phenotypic correlation was decomposed into the ACE components using a correlated factors model and compared it with various sub‐models (AE, CE, and E).^[^ ^58^ ^]^ The optimal model referring to Chi‐square changes, AIC, and the parsimony principle was selected.^[^ ^54^, ^55^ ^]^ The significance of the bivariate genetic correlation was determined by the Bonferroni‐corrected threshold (Bonferroni correction *p* < 0.05 per number of ROIs).

##### Candidate Genes – Brain – Behavior Association Analysis

In the current study, it was focused on *DRD2/ANKK1‐Taq Ia (rs1800497), DRD2 C957T (rs6277)*, *rs2283265*, and *TPH2 (rs4570625)* polymorphism. SNP genotyping was conducting for all participants (see Material S5, Supporting Information for procedures). It was examined whether this SNP distribution followed Hardy–Weinberg Equilibrium by comparing with the SNP distribution of large East Asian populations from 1000 Genomes. The hierarchical linear models (HLMs) (estimating the random intercept of the family) were further used to identify dopaminergic and serotonergic SNPs simultaneously related to the learning rate and brain activity modulated by PE. For SNPs which could significantly predict both the learning rate and brain activation, it was further tested for the mediating role of brain activity between SNPs and the learning rate.

##### Regional Gene Expression in SMA/mSFG

The AHBA dataset provided brain‐wide gene expressions with 3702 spatially distinct samples collected from six postmortem brains.^[^ ^59^ ^]^ The preprocessing procedure was carried out according to the previously published guide:^[^ ^60^ ^]^ 1) each probe was annotated to genes using the Re‐annotator toolbox;^[^ ^61^ ^]^ 2) probes were filtered and the probe which did not exceed the background signal in 50% of all samples across 6 subjects would be excluded; 3) probe selection, selecting the probe with highest differential stability across 6 subjects as the representative probe for each gene; 4) considering the limited samples located in the right hemisphere, the samples would be mirrored to the opposite hemisphere and then the samples with mean distance to ROI less than 2 mm would be assigned to the target region, which simultaneously had significant phenotypic and genetic correlation with the learning rate (i.e. supplementary motor area/ medial superior frontal gyrus (SMA/mSFG)); 5) for each sample, the gene expression was normalized according to the scaled robust sigmoid transformation; 6) genes were filtered based on differential stability across 6 subjects. The whole process was implemented with the Abagen toolbox.^[^ ^62^ ^]^ Here the SMA/mSFG was the ROI and the mean gene expression was calculated as the regional expression level for each gene. The top 5% (*n*  =  785) highly expressed genes were identified as the prominently expressed genes.

##### Enrichment Analysis

For each given gene list, pathway, and process enrichment analyses were carried out by Metascape analysis (https://metascape.org/gp/index.html#/main/step1), with the following ontology sources: KEGG Pathway, GO Biological Processes, Reactome Gene Sets, Canonical Pathways, CORUM, WikiPathways, and PANTHER Pathway. All genes in the genome were used as the enrichment background. Terms with a *p*‐value < 0.01, a minimum count of 3, and an enrichment factor >1.5 (the enrichment factor was the ratio between the observed counts and the counts expected by chance) were collected and grouped into clusters based on their membership similarities. More specifically, *p*‐values were calculated based on the cumulative hypergeometric distribution, and *q*‐values were calculated using the Benjamini–Hochberg procedure to account for multiple correction. Kappa scores were used as the similarity metric when performing hierarchical clustering on the enriched terms, and sub‐trees with a similarity of >0.3 were considered a cluster. The most statistically significant term within a cluster was chosen to represent the cluster.

To further capture the relationships between the terms, a subset of enriched terms was selected and rendered as a network plot, where terms with a similarity >0.3 were connected by edges. The terms with the best *p*‐values from each of the 20 clusters, with the constraint that there were no more than 15 terms per cluster and no more than 250 terms in total were selected. The network was visualized using Cytoscape, where each node represented an enriched term and was colored by its cluster ID (Figure 6B).^[^ ^63^ ^]^

#### 2.4.2 Study‐2 (Longitudinal Survey)

##### Predicting Long‐Term Depressive Symptoms

Hierarchical linear model (HLM) was used to examine the effect of learning rate on the total score of the BDI‐II 8 years later, controlling for the random intercept of family.^[^ ^46^ ^]^ Bivariate genetic modeling was performed to examine whether learning rate and depressive symptoms shared a phenotypic correlation and a common genetic correlation. Mediation analysis was also conducted to examine whether *DRD2* polymorphisms would affect depressive symptoms via the mediating effect of the learning rate.

##### Predicting Other Long‐term Adaptation Outcomes

HLM was used to examine the effect of learning rate on other long‐term learning and adaptation outcomes, including psychological resilience and cognitive flexibility.^[^ ^64^, ^65^ ^]^ The effect of learning rate on the trait and state anxiety measured by the state‐trait anxiety inventory was also examined.^[^ ^66^ ^]^

## 3 Results

### 3.1 Generalized Mixed Effects Modeling (GLMM)

In UG, participants played 48 trials in total, 24 with human proposers and 24 with computer proposers, generating the proposer type variable (human vs computer proposers). In each round, the partner distributed money between him/herself and the participant, and the proportion of money distributed to the participant in the total amount is denoted by the split ratio variable. We used the GLMM to investigate the main and interactional effect of proposer type, split ratio, and time on the binomial decision outcome of acceptance or rejection in the UG. We found significant main effects of split ratio (*z *= 34.36, *p *< .001), proposer type (*z *= 8.01, *p *< .001), and trial number (*z *= 3.03, *p *= .003). Participants had a significantly lower acceptance rate toward human proposers than computer proposers (*estimate (SE) *= −0.60 (.07), *z *= −8.13, *p *< .001). We found a significant interaction between the split ratio and proposer type (*z *= −2.22, *p *= .027). The effect of the split ratio was significantly higher in the human than computer proposer condition (*estimate (SE) *= 1.36 (.62), *z *= 2.22, *p *= .027). This suggests a higher fairness sensitivity toward humans than computer proposers, motivating that two separate fairness sensitivity parameters toward human and computer proposers should be specified in the computational model.

We also found a significant trial number by proposer type interaction (*z *= −2.72, *p *= .006). Participants had a larger time effect when facing human proposers than computer proposers (*estimate (SE) *= .015 (.005), *z *= 2.78, *p *= .006). Specifically, the time effect was only significant for human proposers (*estimate (SE) *= .011 (.004), 95%CI = [.004,.019]) and not for computer proposers (*estimate (SE) *= −.003(.004), 95%CI = [−.011,.004]). This suggested that the acceptance rate increases with time when participants face only human proposers, even after controlling for the ratio effect. This might imply the presence of a norm adaptation process specific to the human proposer condition.

### 3.2 Computational Modeling

We quantitatively specified three critical processes (i.e., a value calculation process, a decision process, and a norm adaptation process) in computational modeling and built different candidate norm adaptation models with slight differences in the norm adaption process (see Section 2.4.1). We performed model fitting, model comparison to select the optimal model with the best fit with the experimental data. Among various candidate models depicting the decision process in the UG, model comparison favored Model‐1 to other models as Model‐1 had the lowest LOOIC (Tables S2, S3, Supporting Information). Model‐1 assumed a learning process for only human proposers but not computer proposers, which was consistent with the results of our GLMM analyses. The 95% highest density intervals (HDIs) for group‐level parameters are shown in Tables S2, S4 (Supporting Information). Parameter estimation revealed a higher fairness sensitivity toward humans than computer proposers, echoing model‐free GLMM analyses (95% HDI: [.02,.05]). Posterior prediction check suggested that the simulated response from Model‐1 had a predictive accuracy of 91.21% on the original response, and could capture key characteristics of the original response (Material S6, Supporting Information). Parameter recovery suggested a high correlation between original and recovered parameters (Material S7, Supporting Information).

In the optimal model (Model‐1), we found a high Pearson correlation between the initial fairness norm and fairness sensitivity (*r *= .87). This implied that a high internal consistency between the two indices of the fairness valuation process (i.e., the initial fairness norm and fairness sensitivity). We also found a low Pearson correlation (*r *= −.18) between the initial fairness norm and the learning rate. This implied that the fairness valuation process (indexed by the initial fairness norm and fairness sensitivity) was phenotypically distinct from the learning process (indexed by the learning rate). In the next section, we would investigate the heritability underlying these two distinct processes, respectively.

### 3.3 Heritability Analysis on Behavioral Indicators

The ICC for parameters of Model‐1 and the acceptance rate toward human and computer proposers are shown in **Table** **1**. Comparison of ICC using the Fisher r‐to‐z transformation suggested a (marginally) significantly higher ICC for MZ than DZ for the initial fairness norm, learning rate, fairness sensitivity toward human proposers and acceptance rate toward human proposers. This potentially implied considerable genetic influences on these parameters. We thus conducted univariate heritability analyses exclusively on these parameters.

**Table 1:** Intra‐class correlations, model comparison, and heritability estimates for model parameters and acceptance rates.

Variable	ICC			Model	AIC	*a* ^2^	*c* ^2^	*e* ^2^
	MZ	DZ	Fisher *z*, *p*					
*Initial norm* (*initial fairness norm*)								
	0.62**	0.22	*z *= 2.31, *p *= 0.010	ACE	151.76	0.41 [0.01, 0.59]	.00 [.00, 0.41]	0.59 [0.41, 0.76]
	[0.32, 0.79]	[−0.43, 0.57]		AE	149.76	0.41 [0.18, 0.59]		0.59 [0.41, 0.82]
				E	158.84			1
α_*h*_ (*learning rate ‐ human proposers*)								
	0.57**	0.34	*z *= 1.35, *p *= 0.088	ACE	153.71	0.25 [0.00, 0.55]	0.10 [0.00, 0.46]	0.65 [0.45, 0.88]
	[0.23, 0.76]	[−0.22, 0.64]		AE	151.80	0.36 [0.13, 0.55]		0.64 [0.45, 0.87]
				E	158.84			1
β_*h*_ (*fairness sensitivity ‐ human proposers*)								
	0.54**	0.20	*z *= 1.85, *p *= 0.032	ACE	156.16	0.32 [0.00, 0.53]	0.00 [0.00, 0.37]	0.68 [0.47, 0.92]
	[0.17, 0.74]	[−0.47, 0.56]		AE	154.16	0.32 [0.08, 0.53]		0.68 [0.47, 0.92]
				E	158.84			1
*Accept_h_* (*acceptance rate ‐ human proposers*)								
	0.56**	0.30	*z *= 1.54, *p *= 0.062	ACE	153.45	0.37 [0.00, 0.56]	0.00 [0.00, 0.44]	0.63 [0.44, 0.86]
	[0.22, 0.76]	[−0.29, 0.62]		AE	151.45	0.37 [0.14, 0.56]		0.63 [0.44, 0.86]
				E	158.84			1
β_*c*_ (*fairness sensitivity ‐ computer proposers*)								
	0.53**	0.32	*z *= 1.20, *p *= 0.114					
	[0.15, 0.74]	[−0.26, 0.63]						
*Accept_c_* (*acceptance rate ‐ computer proposers*)								
	0.52**	0.33	*z *= 1.09, *p *= 0.138					
	[0.14, 0.73]	[−0.24, 0.63]						

The optimal models were underlined. Significant ICCs (*p *< 0.001) were denoted by **;

Abbreviations: ACE: genetic (A), shared environmental (C), and non‐shared environmental (E) contributions; AIC: Akaike Information Criteria; DZ: dizygotic pairs; ICC: intraclass correlation; MZ: monozygotic pairs.

The additive genetic effect and non‐shared environment (AE) model had the optimal fit on all variables, indicated by its lowest Akaike Information Criterion (AIC) compared to alternative models (Table 1). Genetic contributions accounted for 37% of the variance in acceptance rate toward human proposers. Additionally, genetic factors contributed to 41% of the variance in the initial fairness norm, 36% of the variance in the learning rate, and 32% of the variance in the fairness sensitivity toward human proposers.

### 3.4 Voxel‐Wise Univariate Genetic Modeling on PE Maps

**Figure 3:**
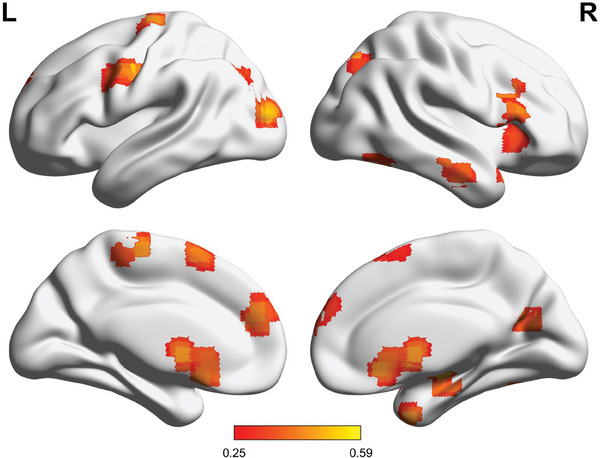
The 18 ROIs extracted from the PPM and their mean heritability across voxels (*n* = 174, cluster size ≥20 and ≥90% posterior probability of having additive genetic effects).

We first selected the optimal model in voxel‐wide univariate genetic modeling. The AE model had the lowest AIC for most voxels (47 819 out of 47 930 voxels). We thus specified the AE model for analyses. We then aggregated the voxel‐wise results by constructing a PPM.^[^ ^12^, ^56^ ^]^ We conducted a model comparison by a Chi‐square test for each voxel between the AE model and the non‐shared environment (E) model to calculate the probability that AE provided a better fit than E. The probability indicated that adding the genetic effect yielded a better fit to the data. Only voxels showing a genetic effect with ≥90% posterior confidence in the PPM were considered of having a significant additive genetic influence.^[^ ^12^, ^56^ ^]^ Aggregating voxel‐wise results, 18 brain regions with cluster size ≥ 20 voxels were identified from the PPM of PE as the ROIs for the followed analyses (**Figure** **3**), which included the medial prefrontal cortex, SMA/mSFG, right anterior insula, lateral prefrontal cortices, temporal regions, posterior parietal cortex, visual cortices, caudate, parahippocampus, and cerebellum. The mean level of genetic effect across voxels for each of the ROIs was shown in Table S8‐1 (Supporting Information). In the next section, we would like to leverage the bivariate genetic modeling approach to further test whether their encoding of PE shows a phenotypic correlation and shares common genetic influences with the learning rate for each of ROIs.

### 3.5 Bivariate Genetic Modeling

We first selected the optimal model in the 18 bivariate genetic models. The AE model had the lowest AIC in 15 out of the 18 bivariate genetic models (Table S8‐2, Supporting Information). In circumstances where AE models were not optimal, neither AE nor the shared environment and non‐shared environment (CE) model showed significant differences with the full additive genetic effect, non‐shared environment, and shared environment (ACE) model. We thus chose the optimal AE model for subsequent bivariate genetic modeling analyses. The phenotypical correlation and common genetic influence between each ROI and the learning rate are shown in Table S8‐3 (Supporting Information).

**Figure 4:**
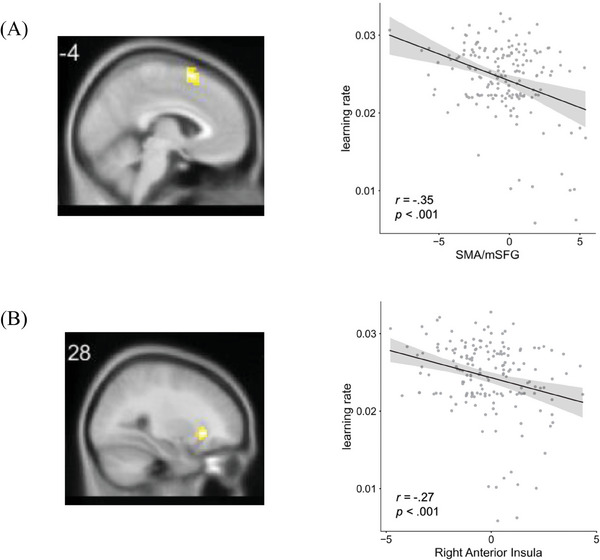
The correlation between the brain activity modulated by norm PE and the learning rate (*n* = 174). A) The correlation between the SMA/mSFG encoding of PE and the learning rate. B) The correlation between the right anterior insula encoding of PE and the learning rate.

Using a Bonferroni‐corrected threshold of *p *< .05/18, we found the parametric estimation value of SMA/mSFG and anterior insula had significant phenotypic correlations with the learning rate, and only SMA/mSFG had a significant genetic correlation with the learning rate. The parametric estimate value for PE in the SMA/mSFG had a significant phenotypic correlation (*r_ph_* = −.34, 95% CI = [−.47, −.20], *p *< .0001) and a significant genetic correlation (*r_g_* = −.61, 95% CI = [−1.00, −.61], *p *< .0001) with the learning rate. The parametric estimate value for PE in the anterior insula also showed a phenotypic correlation with the learning rate (*r_ph_* = −.27, 95% CI = [−.41, −.12], *p *= .0002) but did not show a significant shared genetic influence with the learning rate (*r_g_* = −.36, 95% CI = [−.80,.23], *p *= .172). These results indicated that higher learning rates were associated with a larger magnitude in the encoding of the negative PE in both the SMA/mSFG and the anterior insula (**Figure** **4**A,B), implying that individuals with a higher learning capability had a more sensitive neural representation about how actual split ratios were in violation with the fairness norm.

### 3.6 Candidate Gene–Brain‐Behavior Association Analysis

The frequency distribution of SNPs in the current study had no significant differences with the distribution of SNPs of the East Asian population in the 1000 Genomes Project (Material S9, Supporting Information). We found that the dopaminergic SNPs influenced the learning rate and brain activities modulated by PE encoding. HLMs revealed that *rs1800497, rs2283265*, and their additive score significantly (or marginally) correlated with both the learning rate and the parametric estimate for PE in the SMA/mSFG. Carriers of the *T* allele of *rs1800497* and *rs2283265* were associated with lower learning rates. However, *rs6277* was neither predictive of the learning rate nor the neural activities in the SMA/mSFG (**Table** **2**). We found a unique dopaminergic influence on the neural encoding of PE, as evidenced by the effect of dopaminergic additive score on both the learning rate and the activity of the SMA/mSFG, even after controlling for *TPH2* (**Table** **3**). This finding suggested that dopaminergic genes, rather than *TPH2*, constituted the shared genetic influence affecting both the learning rate and the encoding of PE in neural activities (Table 2).

**Table 2:** The relationship between SNPs, SMA/mSFG activity, and the learning rate.

Regressor	Correlation with the learning rate	Correlation with the SMA/mSFG activity
The influence of dopaminergic genes		
*DRD2 (rs1800497)*	*F*(1, 137.86) = 3.77†, *p *= 0.054 The learning rate of GG was higher than TT/GT homozygotes	*F*(1, 138.87) = **4.92**, *p *= 0.028 GG had a larger magnitude of negative activation in the SMA/mSFG than TT/GT homozygotes
*DRD2 (rs2283265)*	*F*(1, 148.99) = 2.91†, *p *= 0.090 The learning rate of CC was higher than TT/CT homozygotes	*F*(1, 147.85) = 6.85, *p *= 0.010 CC had a larger magnitude of negative activation in the SMA/mSFG than TT/CT homozygotes
Dopaminergic additive score *(rs1800497+ rs2283265)*	*F*(1, 143.93) = 3.57†, *p *= 0.061 TT homozygotes were associated with lower learning rates	*F*(1, 144.09) = 6.17, *p *= 0.014 TT homozygotes were associated with a smaller magnitude of negative activation in the SMA/mSFG
*DRD2 (rs6277)*	*F*(1, 154.24) = 0.46, *p *= 0.501	*F*(1, 150.94) = 1.20, *p *= 0.275
The influence of TPH2		
*TPH2 (rs4570625)*	*F*(1, 127.36) = 0.98, *p *= 0.323	*F*(1, 125.38) = 0.08, *p *= 0.772
The influence of dopaminergic genes and TPH2		
*TPH2* and Dopaminergic additive score	TPH2: *F*(1, 151.73) = 0.25, *p *= 0.616 Dopaminergic additive score: *F*(1, 136.01) = 5.69, *p *= 0.018 Interaction: *F*(1, 165.75) = 2.60, *p *= 0.109	TPH2: *F*(1, 151.31) = 1.02, *p *= 0.315 Dopaminergic additive score: *F*(1, 135.62) = 5.71, *p *= 0.018 Interaction: *F*(1, 165.44) = 0.07, *p *= 0.793

Significant results are bolded. Marginal significant results are marked with †.

**Table 3:** The relationship between SNPs and the initial fairness norm and fairness sensitivity.

Regressor	Correlation with the initial fairness norm	Correlation with the fairness sensitivity
The influence of dopaminergic genes		
*DRD2 (rs1800497)*	*F*(1, 141.41) = 4.71, *p *= 0.032 The initial fairness norm of TT and GT were higher than GG homozygotes	*F*(1, 133.42) = 6.33, *p *= 0.013 The fairness sensitivity of TT and GT was higher than GG homozygotes
*DRD2(rs2283265)*	*F*(1, 153.96) = 3.83†, *p *= 0.052 The initial fairness norm of TT and CT were higher than CC homozygotes	*F*(1, 145.49) = 6.06, *p *= 0.015 The fairness sensitivity of TT and CT was higher than CC homozygotes
Dopaminergic additive score *((rs1800497+ rs2283265)*	*F*(1, 148.43) = 4.42, *p *= 0.037 TT homozygotes were associated with higher fairness norms	*F*(1, 139.65) = 6.48, *p *= 0.012 TT homozygotes were associated with higher fairness sensitivity
*DRD2 (rs6277)*	*F*(1, 155.95) = 0.05, *p *= 0.816	*F*(1, 149.07) = 0.08, *p *= 0.775
The influence of *TPH2*		
*TPH2 (rs4570625)*	*F*(1, 149.21) = 6.75, *p *= 0.010 The initial fairness norm of GG and GT was higher than TT homozygotes.	*F*(1, 143.08) = 2.69, *p *= 0.103
The influence of dopaminergic genes and TPH2		
*TPH2* and Dopaminergic additive score	TPH2: *F*(1, 153.25) = 7.30, *p *= 0.008 Dopaminergic additive score: *F*(1, 137.68) = 5.76, *p *= 0.018 Interaction: *F*(1, 166.63) = 0.33, *p *= 0.566	TPH2: *F*(1, 146.94) = 3.77^†^, *p *= 0.054 Dopaminergic additive score: *F*(1, 130.71) = 7.30, *p *= 0.008 Interaction: *F*(1, 162.60) = 0.34, *p *= 0.561

Significant results are bolded. Marginal significant results are marked with †.

**Figure 5:**
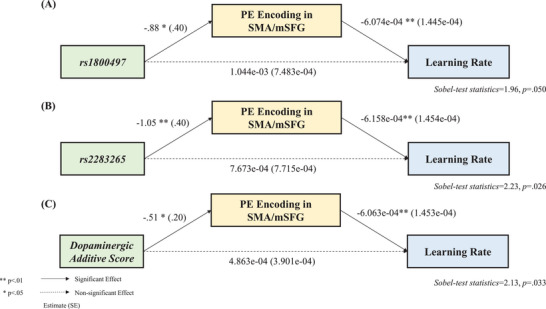
The gene–brain‐behavior mediation models (*n* = 174). A) The mediation of the SMA/mSFG encoding of PE on the relationship between *rs1800497* and the learning rate. B) The mediation of the SMA/mSFG encoding of PE on the relationship between *rs2283265* and the learning rate. C) The mediation of the SMA/mSFG encoding of PE on the relationship between dopaminergic additive scores and the learning rate.

We further found a significant mediation effect of the SMA/mSFG activity on the relationship between (1) *rs1800497* and the learning rate (*sobel‐test statistics *= 1.96, *p *= .050) (**Figure** **5**A), (2) *rs2283265* and the learning rate (*sobel‐test statistics *= 2.23, *p *= .026) (Figure 5B), and (3) dopaminergic additive score and the learning rate (*sobel‐test statistics *= 2.13, *p *= .033) (Figure 5C). These implied that dopaminergic SNPs modulated the norm adaptation capability via SMA/mSFG encoding of PE.

Additionally, we found that distinct genetic contributions underlie the norm adaptation versus the norm fairness valuation system. HLM revealed that the contribution of *rs1800497, rs2283265*, and *rs4570625* to the initial fairness norm was significant or marginally significant both when they were entered separately and together (Table 3). Carriers of the *T* allele of *rs1800497* and *rs2283265* were associated with stricter fairness norms. Carriers of the *G* allele of *rs4570625* were associated with stricter fairness norms. Serotonergic and dopaminergic SNPs contributed to a portion of the unique variance to the initial fairness norm (Table 3). We derived a similar finding for the other indicator of the fairness valuation system, namely the fairness sensitivity (Material S10, Supporting Information).

### 3.7 Gene Enrichment Analysis on Genes Prominently Expressed in the SMA/mSFG

**Figure 6:**
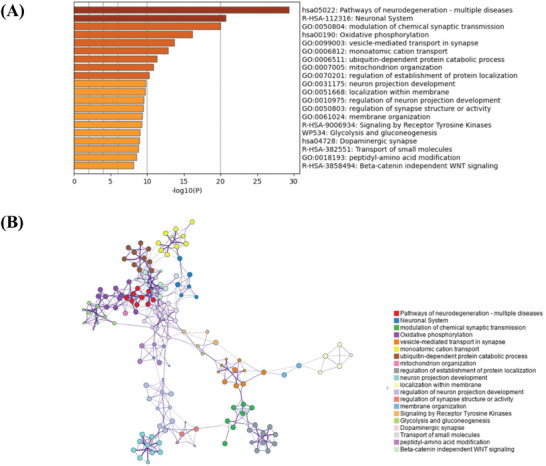
Metascape enrichment analysis. A) Metascape enrichment analysis on top expressed genes in the SMA/mSFG. The *p*‐value was corrected by the FDR and the threshold was set 0.05. Here the enriched terms revealed the possible biological functions that dominantly expressed genes located in SMA/mSFG could be involved with. B) Metascape enrichment network visualization of top 20 genes. The enrichment network visualization shows the intra‐cluster and inter‐cluster similarity of the enriched terms.

The gene enrichment analysis was performed to understand the possible pathways and biological functions that the highly expressed genes located in SMA/mSFG could be involved with. Here the top 5% expressed genes (*n* = 785) located in the SMA/mSFG region were identified as key genes prominently expressed in this region. The gene ontology (GO) biological processes and Kyoto Encyclopedia of Genes and Genomes (KEGG) pathways related to the key gene list were aligned using the Metascape online toolbox. After correcting for enrichment terms (*p_FDR_* < .05), and deleting discrete enrichment clusters, the top‐20 significant GO biological processes and KEGG pathways included the dopaminergic enrichment synapses (*hsa04728*), the modulation of chemical synaptic transmission (*GO: 00 50804*), and the vesicle‐mediated transport in the synapse (*GO: 00 99003*) (**Figure** **6**A). This indicated that the gene activity related with chemical synaptic transmission, especially dopaminergic pathways, and relevant biological processes, was highly active in SMA/mSFG, which potentially explained how gene activity shaped the functionality of this region. The Metascape enrichment network visualization (Figure 6B) clustered the enriched terms and further revealed that dopaminergic synapse took the hub spot of the network, emphasizing again the important role that dopamine‐related gene activity played in the SMA/mSFG.

### 3.8 Predicting Long‐Term Mental Health Outcomes

Long‐term depressive symptoms were measured by the total score of the Beck Depressive Inventory (BDI‐II) (*Mean* (*SD*) = 8.55 (8.67)) (see Material S11, Supporting Information for its density distribution). The learning rate was predictive of depressive symptoms measured by the total score of the BDI‐II (*F*(1118) = 4.67, *p* = .033) eight years later. The learning rate was still predictive of depressive symptoms eight years later after controlling for the baseline depressive scores measured at the time of recruitment (*F*(1114) = 4.56, *p* = .035). The density distribution of the total score of the BDI‐II (*Mean* (*SD*) = 6.38 (5.15)) at baseline was shown in Material S11 (Supporting Information). Bivariate genetic modeling within the optimal AE model further revealed that the two variables shared a significant phenotypic correlation (*r_ph_*  =   −.22,  95% *CI*  =  [−.40,   −.02] ,  *p*  =  .023) and a marginally significant common genetic basis (*r_g_*  =   −.41,  95% *CI*   =  [−.80,  .01] ,  *p*  =  .050), with depressive symptoms showing a high level of heritability (67%, 95%*CI* = [36%, 83%]). Moreover, we found the marginal significant mediation effect of learning rate between *DRD2* gene polymorphisms and depressive symptoms (*sobel‐test statistics* = −1.36, *p* = .087). That is, *DRD2* gene polymorphisms could enhance norm adaptation capability (*estimate* (*SE*) = 6.86e‐4, (3.94e‐4), *p* = .084), which in turn buffer against the occurrence of depressive symptoms 8 years later *(estimate* (*SE*) = −351.72 (161.055), *p* = .031).

The learning rate was marginally significantly predictive of other long‐term learning and adaptation outcomes, including psychological resilience (CD‐RISC) eight years later (*F*(1, 117.9) = 3.88, *p  *= .051), and the alternatives dimensional score (i.e., the adaptive ability to have multiple alternative explanations for life events) of the cognitive flexibility inventory eight years later (*F*(1, 117.0) = 3.30, *p *= .072), controlling for monozygotic/dizygotic pairs, gender and the random intercept of family. These results provide evidence for the external validity of the learning rate extracted from the model. However, the learning rate was not related to trait anxiety (*F*(1, 117.0) = .41, *p *= .521) or state anxiety (*F*(1, 117.3) = .66, *p *= .418).

## 4 Discussion

The current research conducted a series of analyses across two studies to investigate the heritability of norm adaptation ability, its underlying neural mechanisms, and its association with long‐term mental health outcome. We also explored the *DRD2* and serotonergic genetic polymorphisms underlying these processes. In Study‐1 (fMRI experimental study), we found a moderate level of heritability underlying both the overall acceptance rate and sub‐processes extracted by computational modeling, including 1) norm adaptation process, denoted by the learning rate, and 2) fairness valuation process, denoted by the norm strictness (initial fairness norm) and fairness sensitivity. Only dopaminergic polymorphisms *(rs1800497, rs2283265, and their additive scores)* influenced the learning rate, which could be distinct from the serotonergic and dopaminergic genetic underpinnings of the fairness valuation process. Among the 18 identified regions modulated by PE and showing a credible genetic influence via the univariate genetic modeling approach, we further found that the anterior insula had a significant phenotypic correlation but a non‐significant shared genetic correlation with the learning rate, whereas the SMA/mSFG showed both a significant phenotypic correlation and a shared genetic correlation with the learning rate. The dopaminergic *DRD2* polymorphisms simultaneously correlated with the learning rate and the SMA/mSFG encoding of norm PE. What is more, the SMA/mSFG encoding of norm PE mediated the relationship between *DRD2* polymorphisms and the learning rate. Genes associated with the dopaminergic enrichment synapses (*hsa04728*), the modulation of chemical synaptic transmission (*GO: 00 50804*), and the vesicle‐mediated transport in the synapse (*GO: 00 99003*) were highly expressed in the SMA/mSFG, which provided further evidence on the dopamine‐related gene activity in this region. In Study‐2 (longitudinal survey), we found that the norm adaptation capability and long‐term mental health (i.e., self‐reported depression) shared both phenotypic correlation and common genetic influences, and *DRD2* influenced mental health via the mediating role of norm adaptation capability.

Our current study elucidated, for the first time, a moderate level of heritability underlying the two crucial and distinct processes involved in fairness decisions, namely the fairness valuation process and the norm adaptation process. In this study, the initial fairness norm and fairness sensitivity represented a similar underlying construct in the fairness valuation process, displaying a high correlation between them (*r *= .87). Conversely, the initial fairness norm appeared phenotypically distinct from the learning rate, as indicated by a low Pearson correlation (*r *= −.18) in our study, echoing the findings of Gu, et al.^[^ ^13^ ^]^ The fairness valuation process was subject to the influence of dopaminergic SNPs and serotonergic SNP, whereas the norm adaptation process was solely influenced by dopaminergic SNPs, consistent with previous observations of dopaminergic modulation of learning (Bogacz, 2020). These results provide further support for the distinct nature of these two processes.

Furthermore, we found that individuals with varying learning rates showed differences in the strength of norm PE encoding in the anterior insula. That is, individuals with a faster speed of norm learning had a larger magnitude of negative parametric correlations of the anterior insula activation with norm PE. This implied that individuals with faster learning rates have stronger insula activation related to the encoding of negative PEs. Previous studies have also documented the role of insula in representing error signals along various norm dimensions and in initiating actions or belief modifications to diminish such errors.^[^ ^67^ ^]^ Insula lesions result in failures in initiating actions to adjust the subjective norm.^[^ ^13^ ^]^ Norm enforcement also matters in other economic games, especially the Trust Game (TG). The investor could invest any portion of an initial endowment. The amount would be triple and the trustee could decide to return any portion back to the trustor. The trustor could perceive a violation of the reciprocity norm from the trustee when the trustee returns a minimal proportion, thereby punish the trustee by reducing investment in the following trials. The right anterior insula serves to detect norm deviations in the UG and the TG.^[^ ^68^, ^69^, ^70^ ^]^ This is consistent with the lateralization of our major findings. Previous studies have also documented the activation of the insula upon receiving unfair treatments in the UG and upon the experience of aversive emotions such as sadness, anger, and disgust.^[^ ^67^ ^]^ Our study further suggests that the right anterior insula also encodes norm PE signal and that individuals with varying norm adaptation capabilities differ in the neural encoding of norm PE in this brain region.

The common genetic influence of the encoding of norm PE in the right anterior insula with the learning rate did not reach significance. This might imply that different genetic bases underly learning capability and insula activity. Additionally, our supplementary analyses found a non‐significant influence of dopaminergic SNPs on insula activity (Material S10, Supporting Information). This negative finding can be accounted for functional heterogeneity of insula in emotional appraisal and unfairness representation, which makes it potentially susceptible to the influence of a wider heterogeneous variety of SNPs other than those dopaminergic SNPs modulating the learning rate. Future studies could conduct SNP genotyping on a wider range of genes to search for the SNPs that differentially influence the learning rate and insula activity.

Our findings revealed a phenotypic correlation between the strength of norm PE encoding in the SMA/mSFG and the learning rate. Our finding aligns with Chang and Sanfey^[^ ^71^ ^]^ that the encoding of norm PE is associated with the SMA, and aligns with SMA's function in unfairness appraisal and rejection behavior in the UG.^[^ ^72^, ^73^ ^]^ Our finding is also in accordance with SMA's role in learning, including value computation of options in non‐social probabilistic learning tasks, and in action monitoring and error detection in Simon tasks.^[^ ^74^, ^75^, ^76^ ^]^

Bivariate genetic modeling analysis further revealed that a common dopaminergic genetic influence existed between the learning rate and norm PE encoding in the SMA/mSFG. Furthermore, *DRD2* influences learning ability via the mediating role of the SMA/mSFG encoding of norm PE. Results from the gene enrichment analysis of SMA/mSFG provide additional support for the high dopamine‐related gene activity in this area. Adding to the dopaminergic modulation in the fronto‐striatal encoding of PE signals, our study provides new evidence that the SMA/mSFG is also subjective to dopamine modulation in PE encoding.^[^ ^77^ ^]^

Our study for the first time established a robust phenotypic and genetic correlation between norm adaptation capability and long‐term adaptive consequences on mental health, which illustrates that our dopaminergic‐genetically powered learning capability could indeed grant us a survival advantage in terms of buffering against depression. *DRD2* variants have been identified as biomarkers for MDD and predictors of social withdrawal.^[^ ^18^, ^78^ ^]^ Our study revealed a new mediating path of social adaptation capability, through which *DRD2* influences mental health.

This study has the following limitations. First, our study examined the influence of a limited number of dopaminergic and serotonergic SNPs on the learning rate and the initial fairness norm, as well as the neural correlates of norm learning. We also utilized a relatively simple way to estimate the additive genetic effect, that is, to calculate the additive score of two *DRD2* SNPs (*rs1800497* and *rs2283265*). Although we found complementary evidence about the dopamine‐related gene activity in SMA/mSFG via gene enrichment analysis, future research could conduct genotyping on a wider variety SNPs or conduct GWASs to explore the additive influences of a broader range of genes on the norm learning capability and its neural correlates. Second, the sample size of our study is comparable with other twin studies employing fMRI data.^[^ ^79^, ^80^ ^]^ However, our sample size is still limited compared to other behavioral studies (without fMRI) on the heritability of social norm and fairness appraisal.^[^ ^10^, ^81^, ^82^ ^]^ Future studies could validate the heritability of norm adaptation capability with behavioral experiments with a greater sample size. Third, we used self‐reported measures to acquire current/history of physical/psychiatric diagnoses, current psychiatric medications, family history of psychiatric diagnoses, neurological or metabolic illnesses, and head injuries. As clinical screening using standardized scales at enrollment would provide a more reliable measure on these variables, future studies could adopt a clinical screening process during recruitment to rule out the confounding effects of these variables on adaptation outcomes. Fourth, the self‐reported measure of BDI‐II might bring potential biases to the data. Future studies could use clinical evaluations on depressive symptoms to eliminate such biases. Fifth, this study did not measure potential environmental factors that might interact with genetic predispositions to influence learning capabilities (e.g., social economic status, stress exposure, etc.). Although beyond the scope of the current study, future studies could include a broader measure of environmental factors and further investigate the mechanism through which genes influence learning capabilities. Sixth, this study did not employ an additional sample to formally test for the reproducibility of our findings from the genetic and fMRI analyses. Future studies could replicate our findings in another independent twin sample.

## 5 Conclusion

In conclusion, our study reveals that heritability is a non‐negligible driving force behind learning of social norms and its neural basis, which enhances the rapid acquisition of adaptive values across generations, granting us an evolutionary advantage in changing environments in terms of maintaining long‐term mental health. As norm adaptation constitutes one important aspect of norm evolution dynamics, these findings for the first time elucidate the critical role of heritability in fueling social norm evolution and mental health. As dopamine‐related gene activity underlies norm adaptation, its neural basis, and the occurrence of long‐term depressive symptoms, our study provides potential dopaminergic genetic markers (i.e., carriers of the T allele of *rs1800497* and *rs2283265)* for identifying individuals at a higher risk of having social mal‐adaptations and depressive symptoms. This genotype biomarker could enable the early identification of high‐risk individuals and the timely administration of intervention strategies targeted at enhancing social adaptation capabilities and reducing depression. Furthermore, our study revealed that the anterior insula and the SMA/mSFG encoding of norm PE may serve as heritable neural signatures of norm adaptation capability. Future studies could further explore the role of these two regions in encoding violations of a broader range of social norms. To elucidate the causal relationship between dopamine levels and norm adaptation capabilities, future studies could directly manipulate dopamine levels to observe corresponding changes in fairness adaptation and its neural activities.

## Conflict of Interest

The authors declare no conflict of interest.

## Author Contributions

Y.J. dealt with conceptualization, methodology, software, formal analysis, validation, writing the original draft, and visualization. D.Z. dealt with conceptualization, methodology, software, formal analysis, investigation, data curation, writing the review, and editing. R.G. dealt with conceptualization, writing the review and editing, and supervision. Q.F. dealt with conceptualization, visualization, writing the review, and editing. M.D. dealt with conceptualization, writing the review and editing, and supervision. C.W. dealt with conceptualization, methodology, formal analysis, visualization, writing the review and editing, and supervision. X.L. dealt with conceptualization, data curation, writing the review and editing, and supervision. J.C. dealt with conceptualization, data curation, writing the review and editing, and supervision. Y.H. dealt with conceptualization, data curation, writing the review, and editing. Y.Z. dealt with conceptualization, methodology, software, validation, resources, writing the review and editing, supervision, project administration, and funding acquisition

## Supporting information



Supporting Information

## Data Availability

The data that support the findings of this study are available from the corresponding author upon reasonable request.
