# Supplementary material for: Substantial Heritability Underlies Fairness Norm Adaptation Capability and its Neural Basis
Source: Adv Sci (Weinh). 2024 Dec 16;12(9):2411070. doi: 10.1002/advs.202411070 (PMC11884581; doi:10.1002/advs.202411070)
Supplement: Supplementary file 1 — Supporting Information [file ADVS-12-2411070-s001.docx]

Supplementary Material for

Substantial heritability underlies fairness norm adaptation capability and its neural basis

Yuening Jin^a,b^, Dang Zheng ^a,c^, Ruolei Gu^a,b^, Qingchen Fan^a,b^, Martin Dietz^d^, Changshuo Wang^a,e,f^, Xinying Li^g,b^, Jie Chen^g,b^, Yuanyuan Hu^a,b^, Yuan Zhou^a,b,h^

^a^ CAS Key Laboratory of Behavioral Science, Institute of Psychology, Chinese Academy of Sciences, Beijing 100101, China.

^b^ Department of Psychology, University of Chinese Academy of Sciences, Beijing 100049, China.

^c^ Department of Early Childhood Education, China National Children’s Center, Beijing 100035, China

^d^ Center of Functionally Integrative Neuroscience, Institute of Clinical Medicine, Aarhus University, Universitetsbyen 3, 8000 Aarhus C, Denmark

^e^ Sino-Danish Center, University of Chinese Academy of Sciences, Beijing 100049, China

^f^ Brainnetome Center, Institute of Automation, Chinese Academy of Sciences, Beijing 100190, China

^g^ CAS Key Laboratory of Mental Health, Institute of Psychology, Chinese Academy of Sciences, Beijing 100101, China

^h^ The National Clinical Research Center for Mental Disorders & Beijing Key Laboratory of Mental Disorders, Beijing Anding Hospital, Capital Medical University, Beijing, China.

**Table of Contents**

**S1 A full list of proposed splits in the experiment**

**S2 Candidate models**

**S3 Procedures for model recovery and parameter recovery**

**S4 fMRI data acquisition and preprocessing steps**

**S5 SNP genotyping procedures**

**S6 Model recovery results**

**S7 Parameter recovery results**

**S8 Genetic modeling of neuroimaging data**

**S9 Testing for Hardy-Weinberg equilibrium**

**S10 The influence of dopaminergic and serotonergic SNPs on the PE encoding in the anterior insula**

**S11 Density distribution of the total score of BDI-II**

**S1 A full list of proposed splits in the experiment**

Table S1-1 A full list of proposed splits in the experiment

| Offer to Self  (Proposer) | Offer to the Participant  (Responder) | Percentage to the Participant |
| --- | --- | --- |
| 9 | 9 | 50% |
| 11 | 10 | 48% |
| 13 | 11 | 46% |
| 12 | 9 | 43% |
| 14 | 10 | 42% |
| 17 | 11 | 39% |
| 15 | 9 | 38% |
| 18 | 10 | 36% |
| 22 | 11 | 33% |
| 20 | 9 | 31% |
| 24 | 10 | 29% |
| 29 | 11 | 28% |
| 26 | 9 | 26% |
| 32 | 10 | 24% |
| 39 | 11 | 22% |
| 36 | 9 | 20% |
| 46 | 10 | 18% |
| 58 | 11 | 16% |
| 56 | 9 | 14% |
| 74 | 10 | 12% |
| 99 | 11 | 10% |
| 104 | 9 | 8% |
| 157 | 10 | 6% |
| 264 | 11 | 4% |

**S2 Candidate models**

Table S2-1 A list of all candidate models

| Model No. | A norm learning process for human proposers? | A norm learning process for computer proposers? | Fairness norms towards human and computer proposers update together or separately? | Same or different initial fairness norm for human versus computer proposers? | Free parameters |
| --- | --- | --- | --- | --- | --- |
| 1 | Yes | No | NA | Same | $Initial Norm$, $\alpha_{h}$, $\beta_{h}$, $\beta_{c}$, $\tau$, $\xi$ |
| 2 | Yes | Yes, same as human | Separately | Same | $Initial Norm$, $\alpha$, $\beta_{h}$, $\beta_{c}$, $\tau$, $\xi$ |
| 3 | Yes | Yes, same as human | Together | Same | $Initial Norm$, $\alpha$, $\beta_{h}$, $\beta_{c}$, $\tau$, $\xi$ |
| 4 | Yes | Yes, different from human | Separately | Same | $Initial Norm$, $\alpha_{h}$, $\alpha_{c}$, $\beta_{h}$, $\beta_{c}$, $\tau$, $\xi$ |
| 5 | Yes | Yes, different from human | Together | Same | $Initial Norm$, $\alpha_{h}$, $\alpha_{c}$, $\beta_{h}$, $\beta_{c}$, $\tau$, $\xi$ |
| 6 | Yes | No | NA | Different | ${Initial Norm}_{h}$, ${Initial Norm}_{c}$, $\alpha_{h}$, $\beta_{h}$, $\beta_{c}$, $\tau$, $\xi$ |
| 7 | Yes | Yes, same as human | Separately | Different | ${Initial Norm}_{h}$, ${Initial Norm}_{c}$, $\alpha$*,* $\beta_{h}$, $\beta_{c}$, $\tau$, $\xi$ |
| 8 | Yes | Yes, different from human | Separately | Different | ${Initial Norm}_{h}$, ${Initial Norm}_{c}$, $\alpha_{h}$, $\alpha_{c}$, $\beta_{h}$, $\beta_{c}$, $\tau$, $\xi$ |

Note: The candidate models have walked through all possible combinations of norm learning process for human/computer proposers, updating style, and initial fairness norm. There do not exist models which specify different initial fairness norms for human and computer proposers and at the same time assume norms are updating together.

Table S2-2 Prior distributions and ranges for group-level and individual-level free parameters

| Level | Priors and Ranges |
| --- | --- |
| Group-level | $\mu_{initial norm}\sim Normal(0,0.5)$  $\mu_{\alpha}, \mu_{\beta},\mu_{\tau}, \mu_{\xi}\sim Normal(0,1)$  ${\sigma^{2}}_{initial norm},{\sigma^{2}}_{\alpha},{\sigma^{2}}_{\beta},{\sigma^{2}}_{\tau},{\sigma^{2}}_{\xi}\sim Inv-Gamma(1,1)$  $initial norm\in[0,0.5]$  $\alpha\in[0,1]$  $\beta\in[0,1]$  $\tau\in[0,5]$  $\xi\in[0,1]$ |
| Individual-level | ${initial norm}_{i}\sim Normal (\mu_{initial norm},{\sigma^{2}}_{initial norm})$  $\alpha_{i}\sim Normal (\mu_{\alpha},{\sigma^{2}}_{\alpha})$  $\beta_{i}\sim Normal (\mu_{\beta},{\sigma^{2}}_{\beta})$  $\tau_{i}\sim Normal (\mu_{\tau},{\sigma^{2}}_{\tau})$  $\xi_{i}\sim Normal (\mu_{\xi},{\sigma^{2}}_{\xi})$  ${initial norm}_{i}\in[0,0.5]$  $\alpha_{i}\in[0,1]$  $\beta_{i}\in[0,1]$  $\tau_{i}\in[0,5]$  $\xi_{i}\in[0,1]$ |

Table S2-3 The LOOIC for all candidate models

|  | M1 | M2 | M3 | M4 | M5 | M6 | M7 | M8 |
| --- | --- | --- | --- | --- | --- | --- | --- | --- |
| LOO | 5090.3  (186.0) | 5103.2  (184.4) | 5132.4  (184.0) | 5166.6  (184.1) | 5226.2  (184.3) | 5116.3  (185.4) | 5149.4  (186.0) | 5224.9  (185.3) |
| -M1 | -- | 6.5  (6.7) | 21.0  (8.3) | 38.1  (7.6) | 68.0  (14.6) | 13.0  (12.2) | 29.5  (13.1) | 67.3  (16.3) |

Table S2-4 The 95% HDIs for group-level parameter estimations

| $Initial$  $Norm$ | $\alpha_{h}$ | $\beta_{h}$ | $\beta_{c}$ | $\tau$ |
| --- | --- | --- | --- | --- |
| 0.29  [0.26, 0.31] | 0.025  [0.02, 0.03] | 0.91  [0.90, 0.93] | 0.88  [0.86, 0.89] | 3.07  [2.87, 3.28] |

**S3 Procedures for model recovery and parameter recovery**

To perform model recovery, we simulated trial-wise responses using individual-level parameter estimates in the same UG task structure used in the study. We used GLMM to examine whether simulated data could capture key characteristics in the original responses including (1) a higher acceptance rate towards computer than human proposers; (2) a higher fairness sensitivity towards human proposers than computer proposers; (3) a higher time effect for human than computer proposers, and the time effect is significant only for human proposers.

To perform parameter recovery for the winning model, we first simulate trial-wise responses using individual-level parameter estimates in the same UG task structure used in the study. We then derive parameter estimates with simulated data. We then calculated the Pearson correlation between individual-level original and recovered parameters. We repeated the above procedure 20 times to derive 20 correlation indices.

**S4 fMRI acquisition and data preprocessing steps**

Structural images were acquired with a magnetization-prepared rapid acquisition gradient-echo (MPRAGE) sequence with repetition time (TR) = 6.896 ms, echo time (TE) = 2.992 ms, flip angle = 8°, inversion time (TI) = 450 ms, slice thickness = 1 mm (no gap), field of view (FoV) = 256 * 256 * 176 mm. Functional images were acquired with an echo planar imaging sequence with TR = 2000 ms, TE = 30 ms, flip angle = 70°, matrix = 64 * 64, FoV = 220 mm * 220 mm, slice thickness = 3.5 mm, slice gap = 0.5mm, 33 axial slices for each brain volume. Scanning consisted of 2 sessions, each of which lasted for approximately 8 minutes. The experimental stimuli were presented on a personal computer and were then projected onto a screen with an LCD projector. Participants viewed the screen through a mirror installed on the MRI head coil. Volume acquisition was synchronized to stimulus presentation on E-prime. After image preprocessing using Statistical Parametric Mapping (SPM12; Wellcome Department of Cognitive Neurology, Institute of Neurology, London, England) and DPABI v3.0 ^1^, we found 6 participants with maximum head motion exceeding 3.0 mm or 3.0°. We excluded these individuals and their twin siblings.

We included the following preprocessing steps. First, functional images were slice-timing corrected and spatially realigned with a six-parameter affine transformation. 6 participants with >3mm maximum displacement or >3° angular rotation in any of the x, y, or z axis and their twin were excluded. This resulted in 174 participants. Then images were normalization to MNI space and resampled to 3mm isotropic. Then they were smoothed with a Gaussian kernel of 6mm full-width at half maximum.

**S5 SNP genotyping procedures**

We collected saliva from each participant which was stored in a -80°C environment. The Polymerase chain reaction (PCR) for *rs1800497*, *rs2283265*, *rs6277* and *rs4570625* genotyping was conducted with a 5.0 ul PCR reaction in total, with 1.0 ul of DNA, 10X PCR Buffer, 0.1 ul of HotstarTaq, 1.0 ul of 500nM each Primer mix (*rs1800497* forward primer: 5ʹ- ACGTTGGATGACACAGCCATCCTCAAAGTG-3ʹ; reverse primer: 5ʹ- ACGTTGGATGTGTGCAGCTCACTCCATCCT-3ʹ; *rs2283265* forward primer: 5ʹ-ACGTTGGATGATGAGGAAACAGGCTCATAG-3ʹ; reverse primer: 5ʹ- ACGTTGGATGTCAGATCCTGTCACTGACAC-3ʹ; *rs6277* forward primer: 5ʹ-ACGTTGGATGCTTTGGCATGCCCATTCTTC-3ʹ; reverse primer: 5ʹ-ACGTTGGATGTCCCACCATGGTCTCCACAG-3ʹ; *rs4570625* forward primer: 5ʹ-ACGTTGGATGACTCACACATTTGCATGCAC-3ʹ; reverse primer: 5ʹ-ACGTTGGATGACTCATTGACCAACTCCATT-3ʹ), and 0.1 ul of 25mM each dNTP mix in the GeneAmp® PCR System 9700 Dual 384-Well Sample Block Module. The thermocycling conditions included (1) initial denaturation for 15s at 94°C; (2) 45 cycles of denaturation at 94°C for 20 seconds, annealing at 56°C for 30 seconds, and an extension at 72°C for 60 seconds; (3) a final extension at 72°C for 3 minutes and stored at 4°C forever. Then we administered SAP enzyme treatment to degrade dNTPs used in the PCR to ensure extension reaction only extends by one base. Then we conducted single base extension reaction using extension primers. After resin desalting purification, we conducted chip spotting with Nanodispenser SpectroCHIP. Then we conducted mass spectrometry detection with the MassARRAY Analyzer Compact. Lastly, we used TYPER 4.0 software to analyze experimental results and finally obtained the genotyping data for each participant.

**S6 Model recovery results**

Figure S5-1 shows the average acceptance rate in each split ratio for human and computer proposers for the original and recovered responses. The predictive accuracy of the winning model (i.e. Model-1) is 91.21%. The simulated responses could well capture key characteristics in the original responses including: (1) a lower acceptance rate towards human than computer proposers (*estimate (SE)* = -1.33 (.13), *z* = -9.53, *p* < .001); (2) a higher fairness sensitivity towards human than computer proposers (*estimate (SE)* = 11.50 (1.33), *z* = 8.62, *p* < .001); (3) a higher time effect for human than computer proposers (*estimate (SE)* = .048 (.010), *z* = 4.95, *p* < .001), and the time effect was only significant for human proposers (*estimate (SE)* = .042 (.007), 95% CI = [.029, .056]) but not computer proposers (*estimate (SE)* = -.005 (.007), 95% CI = [-.019, .008]).

1. Original responses


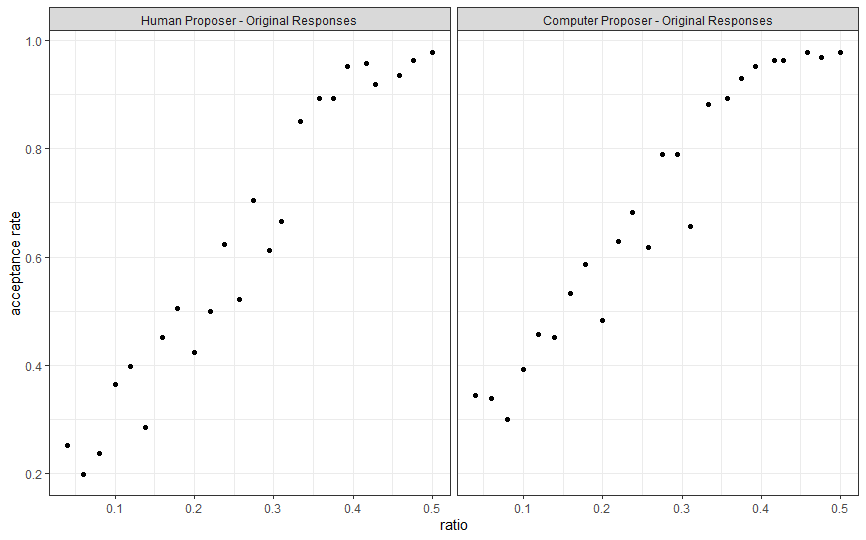


1. Simulated responses


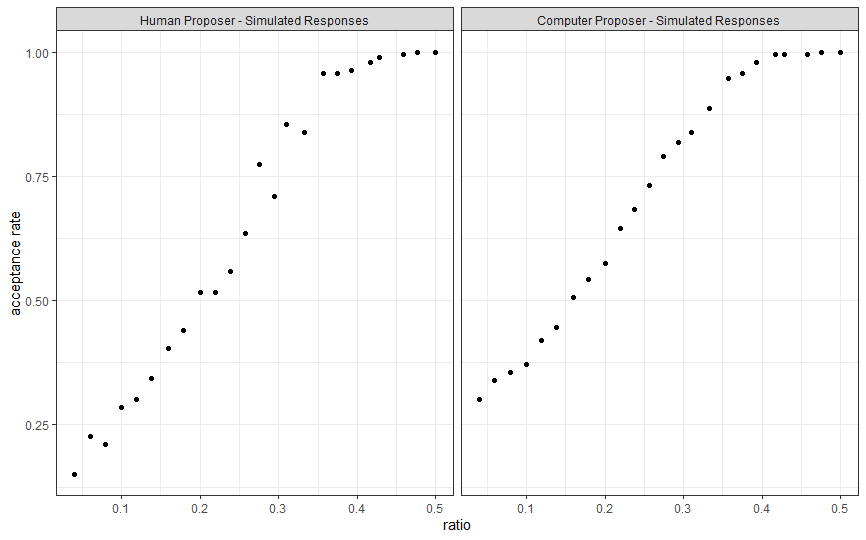


Figure S5-1 The average acceptance rate for human and computer proposers for the (1) original and (2) recovered responses

**S7 Parameter recovery results**

Table S6 Pearson correlation (*r* and *SD*) between original and recovered parameters

|  | $Initial$  $Norm$ | $\alpha_{h}$ | $\beta_{h}$ | $\beta_{c}$ | $\tau$ | $\xi$ |
| --- | --- | --- | --- | --- | --- | --- |
| *r* | .99 | .99 | .98 | .92 | .87 | .68 |
| *SD* | .001 | .002 | .003 | .009 | .017 | .036 |

**S8 Genetic modeling of neuroimaging data**

Table S8-1 Regions of interest (ROIs) with > =90% probability of having a genetic influence.

| No. | Name of ROIs | BA | Cluster size | Peak MNI  Coordinates | | | Average  Heritability  across Voxels |
| --- | --- | --- | --- | --- | --- | --- | --- |
|  |  |  |  | X | Y | Z |  |
| 1 | Medial Prefrontal Cortex (mPFC) | 9,10 | 92 | -9 | 48 | 24 | 0.33 |
| 2 | Supplementary Motor Area (SMA)/Medial Superior Frontal Gyrus (mSFG) | 6 | 47 | -3 | 12 | 60 | 0.31 |
| 3 | Subgenual Anterior Cingulate Cortex (sgACC) | 25 | 52 | 3 | 24 | -9 | 0.34 |
| 4 | Left Precentral Gyrus/ Inferior Frontal Gyrus (IFG) | 6,9 | 54 | -45 | -6 | 36 | 0.34 |
| 5 | Right IFG | 47,45 | 124 | 51 | 18 | -6 | 0.36 |
| 6 | Left Paracentral Lobule/Precentral Gyrus | 6 | 37 | -21 | -21 | 69 | 0.36 |
| 7 | Right Anterior Insula | 13 | 40 | 27 | 24 | -3 | 0.33 |
| 8 | Right Inferior Temporal Gyrus (ITG)/Middle Temporal Gyrus (MTG) | 20,21 | 33 | 69 | -21 | -18 | 0.34 |
| 9 | Right Fusiform Gyrus | 37 | 27 | 36 | -57 | -21 | 0.32 |
| 10 | Left Parahippocampal Gyrus/Uncus | 34 | 20 | -9 | -9 | -24 | 0.42 |
| 11 | Right Parahippocampal Gyrus | 28 | 29 | 21 | -15 | -15 | 0.35 |
| 12 | Right Uncus | 28,36 | 30 | 15 | 6 | -33 | 0.37 |
| 13 | Right Precuneus/Superior Occipital Gyrus (SOG)/Superior Parietal Lobule (SPL) | 7,31,19 | 71 | 27 | -75 | 51 | 0.34 |
| 14 | Left Middle Occipital Gyrus (MOG) | 19,18 | 66 | -36 | -93 | 15 | 0.38 |
| 15 | Left MOG | 19 | 52 | -33 | -72 | 36 | 0.31 |
| 16 | Right Caudate |  | 53 | 6 | 3 | 0 | 0.35 |
| 17 | Left Cerebellum Crus II & I |  | 87 | -33 | -81 | -36 | 0.33 |
| 18 | Right Cerebellum Crus II |  | 27 | 15 | -90 | -39 | 0.37 |

Table S8-2 Results of bivariate genetic analysis between the learning rate and the mean parametric estimate value for PE in the ROIs

| Measure and Model | -2LL | df | AIC | Change from full model | | |
| --- | --- | --- | --- | --- | --- | --- |
|  |  |  |  | $\Delta\chi^{2}$ | $\Delta df$ | $p$ |
| Medial Prefrontal Cortex (mPFC) | | | | | | |
| ACE | 971.97 | 337 | 297.97 |  |  |  |
| AE | 972.19 | 340 | 292.19 | .23 | 3 | .973 |
| CE | 974.74 | 340 | 294.74 | 2.78 | 3 | .428 |
| E | 985.56 | 343 | 299.56 | 13.59 | 6 | .035 |
| Supplementary Motor Area (SMA)/Medial Superior Frontal Gyrus (mSFG) | | | | | | |
| ACE | 949.34 | 337 | 275.34 |  |  |  |
| AE | 949.54 | 340 | 269.54 | .20 | 3 | .978 |
| CE | 951.32 | 340 | 271.32 | 1.98 | 3 | .577 |
| E | 963.97 | 343 | 277.97 | 14.63 | 6 | .023 |
| Subgenual Anterior Cingulate Cortex (sgACC) | | | | | | |
| ACE | 969.65 | 337 | 295.65 |  |  |  |
| AE | 969.85 | 340 | 289.85 | .21 | 3 | .976 |
| CE | 972.27 | 340 | 292.27 | 2.63 | 3 | .453 |
| E | 985.55 | 343 | 299.55 | 15.91 | 6 | .014 |
| Left Precentral Gyrus/ Inferior Frontal Gyrus (IFG) | | | | | | |
| ACE | 937.67 | 335 | 267.67 |  |  |  |
| AE | 937.98 | 338 | 261.98 | .31 | 3 | .958 |
| CE | 945.16 | 338 | 269.16 | 7.49 | 3 | .058 |
| E | 957.16 | 341 | 275.16 | 19.50 | 6 | .003 |
| Right IFG | | | | | | |
| ACE | 967.91 | 337 | 293.91 |  |  |  |
| AE | 968.11 | 340 | 288.11 | .20 | 3 | .978 |
| CE | 971.04 | 340 | 291.04 | 3.13 | 3 | .373 |
| E | 982.16 | 343 | 296.16 | 14.25 | 6 | .027 |
| Left Paracentral Lobule/Precentral Gyrus | | | | | | |
| ACE | 964.10 | 337 | 290.10 |  |  |  |
| AE | 964.86 | 340 | 284.86 | .76 | 3 | .858 |
| CE | 970.42 | 340 | 290.42 | 6.32 | 3 | .097 |
| E | 985.55 | 343 | 299.55 | 21.45 | 6 | .002 |
| Right Anterior Insula (AI) | | | | | | |
| ACE | 956.12 | 337 | 282.12 |  |  |  |
| AE | 957.08 | 340 | 277.08 | .95 | 3 | .812 |
| CE | 959.31 | 340 | 279.31 | 3.19 | 3 | .363 |
| E | 972.49 | 343 | 286.49 | 16.36 | 6 | .012 |
| Right Inferior Temporal Gyrus (ITG)/Middle Temporal Gyrus (MTG) | | | | | | |
| ACE | 969.73 | 337 | 295.73 |  |  |  |
| AE | 970.39 | 340 | 290.39 | .66 | 3 | .882 |
| CE | 973.65 | 340 | 293.65 | 3.92 | 3 | .270 |
| E | 984.82 | 343 | 298.82 | 15.09 | 6 | .020 |
| Right Fusiform Gyrus | | | | | | |
| ACE | 932.39 | 335 | 262.39 |  |  |  |
| AE | 932.75 | 338 | 256.75 | .36 | 3 | .949 |
| CE | 933.64 | 338 | 257.64 | 1.25 | 3 | .740 |
| E | 948.66 | 341 | 266.66 | 16.27 | 6 | .012 |
| Left Parahippocampal Gyrus/Uncus | | | | | | |
| ACE | 967.13 | 337 | 293.13 |  |  |  |
| AE | 967.74 | 340 | 287.74 | .61 | 3 | .894 |
| CE | 972.52 | 340 | 292.52 | 5.39 | 3 | .146 |
| E | 984.47 | 343 | 298.47 | 17.34 | 6 | .008 |
| Right Parahippocampal Gyrus | | | | | | |
| ACE | 895.45 | 335 | 225.45 |  |  |  |
| AE | 895.88 | 338 | 219.88 | .43 | 3 | .935 |
| CE | 901.42 | 338 | 225.42 | 5.97 | 3 | .113 |
| E | 918.98 | 341 | 236.98 | 23.52 | 6 | .001 |
| Right Uncus | | | | | | |
| ACE | 885.98 | 332 | 221.98 |  |  |  |
| AE | 886.54 | 335 | 216.54 | .57 | 3 | .903 |
| CE | 889.90 | 335 | 219.90 | 3.92 | 3 | .270 |
| E | 905.25 | 338 | 229.25 | 19.28 | 6 | .004 |
| Right Precuneus/Superior Occipital Gyrus (SOG)/Superior Parietal Lobule (SPL) | | | | | | |
| ACE | 964.27 | 337 | 290.27 |  |  |  |
| AE | 966.10 | 340 | 286.10 | 1.83 | 3 | .608 |
| CE | 964.29 | 340 | 284.29 | .03 | 3 | .999 |
| E | 980.35 | 343 | 294.35 | 16.08 | 6 | .013 |
| Left Middle Occipital Gyrus (MOG) | | | | | | |
| ACE | 957.05 | 337 | 283.05 |  |  |  |
| AE | 959.17 | 340 | 279.17 | 2.11 | 3 | .549 |
| CE | 957.49 | 340 | 277.49 | .43 | 3 | .933 |
| E | 985.13 | 343 | 299.13 | 28.08 | 6 | .000 |
| Left MOG | | | | | | |
| ACE | 970.31 | 337 | 296.31 |  |  |  |
| AE | 970.53 | 340 | 290.53 | .22 | 3 | .974 |
| CE | 971.75 | 340 | 291.75 | 1.43 | 3 | .697 |
| E | 984.71 | 343 | 298.71 | 14.40 | 6 | .025 |
| Right Caudate | | | | | | |
| ACE | 965.44 | 337 | 291.44 |  |  |  |
| AE | 966.65 | 340 | 286.65 | 1.21 | 3 | .750 |
| CE | 966.57 | 340 | 286.57 | 1.14 | 3 | .769 |
| E | 984.38 | 343 | 298.38 | 18.94 | 6 | .004 |
| Left Cerebellum Crus II & I | | | | | | |
| ACE | 968.63 | 337 | 294.63 |  |  |  |
| AE | 969.64 | 340 | 289.64 | 1.02 | 3 | .797 |
| CE | 969.10 | 340 | 289.10 | .48 | 3 | .923 |
| E | 984.64 | 343 | 298.64 | 16.02 | 6 | .014 |
| Right Cerebellum Crus II | | | | | | |
| ACE | 916.95 | 336 | 244.95 |  |  |  |
| AE | 917.19 | 339 | 239.19 | .24 | 3 | .971 |
| CE | 919.90 | 339 | 241.90 | 2.95 | 3 | .399 |
| E | 933.82 | 342 | 249.82 | 16.87 | 6 | .010 |

Note: ROIs whose optimal models was not AE were marked red.

Table S8-3 Bivariate genetic modeling

| No. | Name of ROIs | Phenotypic correlation with $\alpha_{h}$  ($r_{ph}$ 95% CI, *p*) | Common genetic influence with $\alpha_{h}$  ($r_{g}$ 95% CI, *p*) |
| --- | --- | --- | --- |
|  |  |  |  |
| 1 | mPFC | .01 [-.15, .16]  *p* = .918 | -.26 [-1.00, .33]  *p* = 0.442 |
| 2 | SMA/mSFG | **-.34 [-.47, -.20]**  ***p* < .0001** | **-.61 [-1.00, -.61]**  ***p* < .0001** |
| 3 | sgACC | .01 [-.14, .17]  *p* = .869 | -.29 [-1.00, .24]  *p* = .355 |
| 4 | Left Precentral Gyrus/IFG | -.10 [-.26, .06]  *p* = .205 | -.31 [-.90, -.31]  *p* = .038 |
| 5 | Right IFG | -.13 [-.28, .02]  *p* = 0.088 | -.14 [-.75, -.14]  *p* = .377 |
| 6 | Left Paracentral Lobule/Precentral Gyrus | .00 [-.16, .16]  *p* = .971 | -.41 [-1.00, -.41]  *p* = .006 |
| 7 | Right anterior insula | **-.27 [-.41, -.12]**  ***p* = .0002** | -.36 [-.80, .23]  *p* = .172 |
| 8 | Right ITG/ MTG | .06 [-.09, .22]  *p* = .422 | .16 [-.40, .82]  *p* = .599 |
| 9 | Right Fusiform Gyrus | -.12 [-.27, .03]  *p* = .127 | -.87 [-1.00, -.27]  *p* < .001 |
| 10 | Left Parahippocampal Gyrus/Uncus | -.08 [-.23, .08]  *p* = .343 | -.24 [-.94, .26]  *p* = .438 |
| 11 | Right Parahippocampal Gyrus | .00 [-.16, .15]  *p* = .958 | -.64 [-1.00, -.08]  *p* = 0.006 |
| 12 | Right Uncus | .06 [-.10, .21]  *p* = .477 | -.38 [NA, .14] |
| 13 | Right Precuneus/SOG/SPL | -.17 [-.31, -.02]  *p* = .027 | -.86 [-1.00, -.19]  *p* < .001 |
| 14 | Left MOG | -.04 [-0.20, .11]  *p* = .592 | -.62 [-1.00, -.62]  *p* < .001 |
| 15 | Left MOG | -.07 [-.22, .09]  *p* = .383 | -.14 [-.78, .44]  *p* = .646 |
| 16 | Right Caudate | -.08 [-.23, .08]  *p* = .315 | -.34 [-1.00, .09]  *p* = .223 |
| 17 | Left Cerebellum Crus II & I | -.07 [-.22, .09]  *p* = .395 | -.43 [-1.00, -.43]  *p* = .003 |
| 18 | Right Cerebellum Crus II | -.04 [-.20, .11]  *p* = .598 | -.42 [-1.00, .1]  *p* = .131 |

Note: Significant effects for phenotypic correlation and common genetic influence are shown in bold after Bonferroni correction for the number of ROIs (*p*<0.05/18=0.001).

**S9 Testing for Hardy-Weinberg equilibrium**

Table S9-1 The genotype distribution of SNPs

| *rs1800497* | *rs2283265* | *rs6277* | *rs4570625* |
| --- | --- | --- | --- |
| *TT* (N=24) | *TT* (N=27) | *GT* (N=19) | *GG* (N=45) |
| *GT* (N=86) | *CT* (N=92) | *GG* (N=155) | *GT* (N=87) |
| *GG* (N=63) | *CC* (N=55) |  | *TT* (N=42) |
| *Chi-square* (1) *=* .39*, p*=.533 | *Chi-square* (1) *=*.17, *p*=.683 | *Chi-square* (1) *=*.44, *p*=.505 | *Chi-square* (1) *=*.72, *p*=.396 |

**S10 The influence of dopaminergic and serotonergic SNPs on the PE encoding in the anterior insula**

| Regressor | Correlation with the Anterior Insula |
| --- | --- |
| The Influence of Dopaminergic Genes | |
| *rs1800497* | *F*(1, 135.68)=.23, *p*=.631 |
| *rs2283265* | *F*(1, 144.54)=1.96, *p*=.164 |
| Dopaminergic additive score  *(rs1800497+ rs2283265)* | *F*(1, 140.79)=.94, *p*=.333 |
| *rs6277* | *F*(1, 148.98)=.002, *p*=.959 |
| The Influence of *TPH2* | |
| *rs4570625* | *F*(1, 143.47)=.78, *p*=.378 |
| The Interactional Influence of Dopaminergic Genes and *TPH2* | |
| *TPH2* and Dopaminergic additive score | *TPH2*: *F*(1, 150.59)=.11, *p*=.745  Dopaminergic additive score:  *F*(1, 134.47)=2.56, *p*=.112  Interaction:  *F*(1, 165.25)=3.17, *p*=.080 |

**S11 Density distribution of the total score of BDI-II**


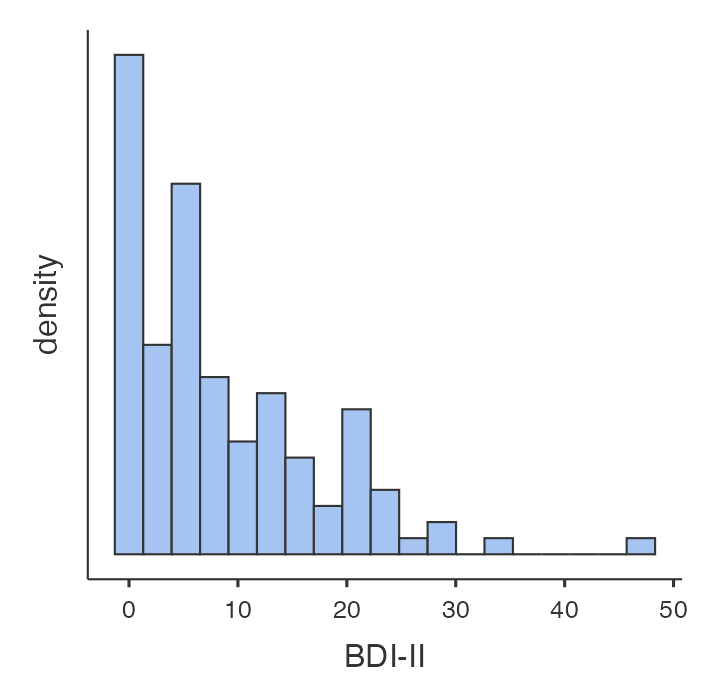


Figure S11-1 The density distribution of the total score of BDI-II eight years later


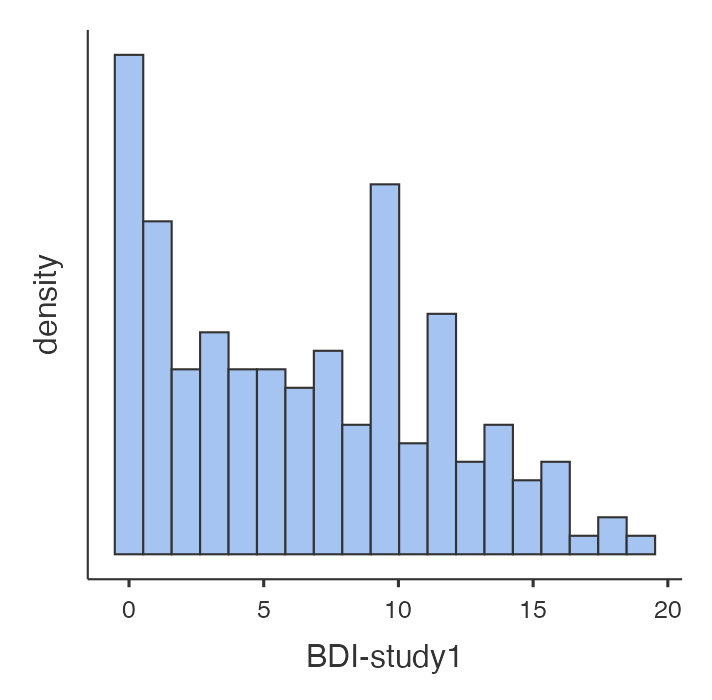


Figure S11-2 The density distribution of the total score of BDI-II at the time of recruitment

**References**

1. Yan, C.-G., Wang, X.-D., Zuo, X.-N., and Zang, Y.-F. (2016). DPABI: data processing & analysis for (resting-state) brain imaging. Neuroinformatics *14*, 339-351. 10.1007/s12021-016-9299-4.
